# Design Study of an Ultrahigh Resolution Brain SPECT System Using a Synthetic Compound-Eye Camera Design With Micro-Slit and Micro-Ring Apertures

**DOI:** 10.1109/TMI.2021.3096920

**Published:** 2021-11-30

**Authors:** Elena Maria Zannoni, Can Yang, Ling-Jian Meng

**Affiliations:** Department of Bioengineering, University of Illinois at Urbana-Champaign, Urbana, IL 61801 USA; Department of Nuclear, Plasma and Radiological Engineering, University of Illinois at Urbana-Champaign, Urbana, IL 61801 USA; Department of Nuclear, Plasma and Radiological Engineering, University of Illinois at Urbana-Champaign, Urbana, IL61801 USA; Beckman Institute for Advanced Science and Technology, University of Illinois at Urbana-Champaign, Urbana, IL 61801 USA

**Keywords:** Brain SPECT, compound-eye camera, gamma camera design, multi-pinhole, solid-state detectors

## Abstract

In this paper, we discuss the design study for a brain SPECT imaging system, referred to as the HelmetSPECT system, based on a spherical synthetic compound-eye (SCE) gamma camera design. The design utilizes a large number ( ~500) of semiconductor detector modules, each coupled to an aperture with a very narrow opening for high-resolution SPECT imaging applications. In this study, we demonstrate that this novel system design could provide an excellent spatial resolution, a very high sensitivity, and a rich angular sampling without scanning motion over a clinically relevant field-of-view (FOV). These properties make the proposed HelmetSPECT system attractive for dynamic imaging of epileptic patients during seizures. In ictal SPECT, there is typically no prior information on where the seizures would happen, and both the imaging resolution and quantitative accuracy of the dynamic SPECT images would provide critical information for staging the seizures outbreak and refining the plans for subsequent surgical intervention.We report the performance evaluation and comparison among similar system geometries using non-conventional apertures, such as micro-ring and micro-slit, and traditional lofthole apertures. We demonstrate that the combination of ultrahigh-resolution imaging detectors, the SCE gamma camera design, and the micro-ring and micro-slit apertures would offer an interesting approach for the future ultrahigh-resolution clinical SPECT imaging systems without sacrificing system sensitivity and FOV.

## I. Introduction

BRAIN-DEDICATED SPECT imaging systems were the first organ-specific SPECT systems being developed since the late 1970s [1]. As the demand for brain SPECT instrumentation with higher spatial resolution, energy resolution and sensitivity continue to rise for studies of neurodegenerative diseases and brain functions, the latest research and commercial systems are based on stationary multi-detector geometries coupled with stationary high-resolution collimators. The G-SPECT-I system [2], [3] uses nine large FOV NaI crystals coupled with 54 focusing pinholes, providing an excellent 2.5 mm spatial resolution with a sensitivity of 415 cps/MBq (0.0415%) when 3 mm-diameter pinhole collimators are used, but in a limited FOV of 10 cm D × 6 cm L. A collaboration between the University of Massachusetts and University of Arizona is developing the AdaptiSPECT-C system [4], [5], a stationary helmet-shaped brain-dedicated SPECT system. The system presents 23 hexagonal detector heads based on NaI(Tl) scintillators and a multi-aperture collimator with temporal shuttering mechanism [6], according to the concept of “Adaptive SPECT” introduced by Barrett *et al*. in [7]. From preliminary performance evaluation, the AdaptiSPECT-C system offers 8 mm spatial resolution with 0.0305% volumetric sensitivity when 2.72 mm-diameter pinhole collimators are used, in a clinically relevant spherical FOV of 21 cm in diameter [4]. Finally, the INSERT project from several groups in Europe developed the first MR-compatible clinical SPECT insert [8]. The system consists of a static ring of 20 MRI compatible CsI(TI) detectors with SiPM readout coupled with a multi-slit-slat collimator [9]. The scanner achieves a spatial resolution of ~8 mm across the FOV (20 cm D × 9 cm L) and sensitivity of ~0.036% [9].

SPECT imaging has been widely used in cardiovascular applications where macroscopic anatomical structures are involved [10]. In the case of cerebrovascular SPECT applications, such as epilepsy or dementia, the microscopic anatomy under evaluation requires spatial resolutions below 10 mm [11], while conventional SPECT cameras can acquire around 8–12 mm. To achieve this, specialized multi-pinhole (or lofthole) geometries, which represent the state-of-the-art in small animal imaging, have been extensively explored and designed for human brain SPECT [12]-[14]. However, due to the small open fractions of the apertures (especially pinholes), the well-known tradeoff between system sensitivity and spatial resolution critically arises. The detection efficiency of such SPECT systems is inevitably reduced to guarantee high spatial resolution, which ultimately limits the efficiency of the systems to collect useful imaging information at reasonable radiation doses.

Many systems mentioned above [1], [3], [9] used conventional cylindrical/annular geometries, which may not represent the most efficient organ-specific design choice. The pioneering work from Rowe *et al*. [15] at University of Arizona introduced the concept of hemispherical system design, where modular cameras were packed in a hemispherical pattern surrounding the patient’s head and coupled with a multiple-pinhole coded aperture. Similarly, the under-development AdaptiSPECT-C system shows a truncated spherical geometry [5], suited for three-dimensional brain imaging. Extensive work has been done to quantify the improved performance of such geometry [4], [5], [16].

In this paper, we present the design of a high-performance dedicated-brain SPECT system, the HelmetSPECT system, that will be potentially used for imaging patients with medically intractable focal epilepsy (MIFE) [17]. MIFE affects almost one third of epileptic patients and requires surgical intervention because it is drug resistant. Nuclear imaging techniques (PET and SPECT) are routinely used for locating the seizure foci and planning the surgical removal, and ictal SPECT imaging is known to be the only non-invasive modality capable of imaging brain activity during an active seizure [18].

The HelmetSPECT system is based on the synthetic compound eye (SCE) camera design [19], and non-conventional micro-slit and micro-ring apertures coupled with modular solid-state detectors. The idea of using semiconductor detectors was previously proposed by Rogulski *et al*. in [20], due to the high spatial resolution and improved energy resolution that such detectors can offer in comparison to scintillator-based detectors, and it has been applied in several preclinical [21]-[23] and clinical systems [24]-[26].

We have carried out a series of simulation studies to evaluate the use of the SCE gamma camera design with a spherical arrangement [27], and to compare the imaging properties offered by the non-conventional apertures with the ones offered by traditional loftholes [28]. The results show that the combination of the SCE camera design and the non-conventional micro-slit and micro-ring apertures would allow for an excellent spatial resolution and high sensitivity over a clinically relevant FOV.

## II. Materials and Methods

### A. The Synthetic Compound Eye (SCE) Gamma Camera Design

The HelmetSPECT system investigated in this study is based on the SCE gamma camera design, which is an extension of the inverted compound-eye design that we proposed and evaluated in [19], [29]. The design is inspired by superposition compound eyes often found in vision systems of small invertebrates [30]. The basic design principles behind the SCE camera are: (*a*) to compose a gamma camera or a complete SPECT system with a large number of closely packed micro-camera-elements (MCE’s) surrounding the object and collectively gathering photons from the FOV with a highly de-magnifying ratio. Each MCE consists of a compact high-resolution gamma-ray detector coupled to an aperture insert which is designed to project an independent view of the object volume; (*b*) to combine different types of MCE’s (e.g., different pinhole sizes and magnification ratios) in order to achieve the desired image quality and offer a great flexibility in imaging.

**Fig. 1.**
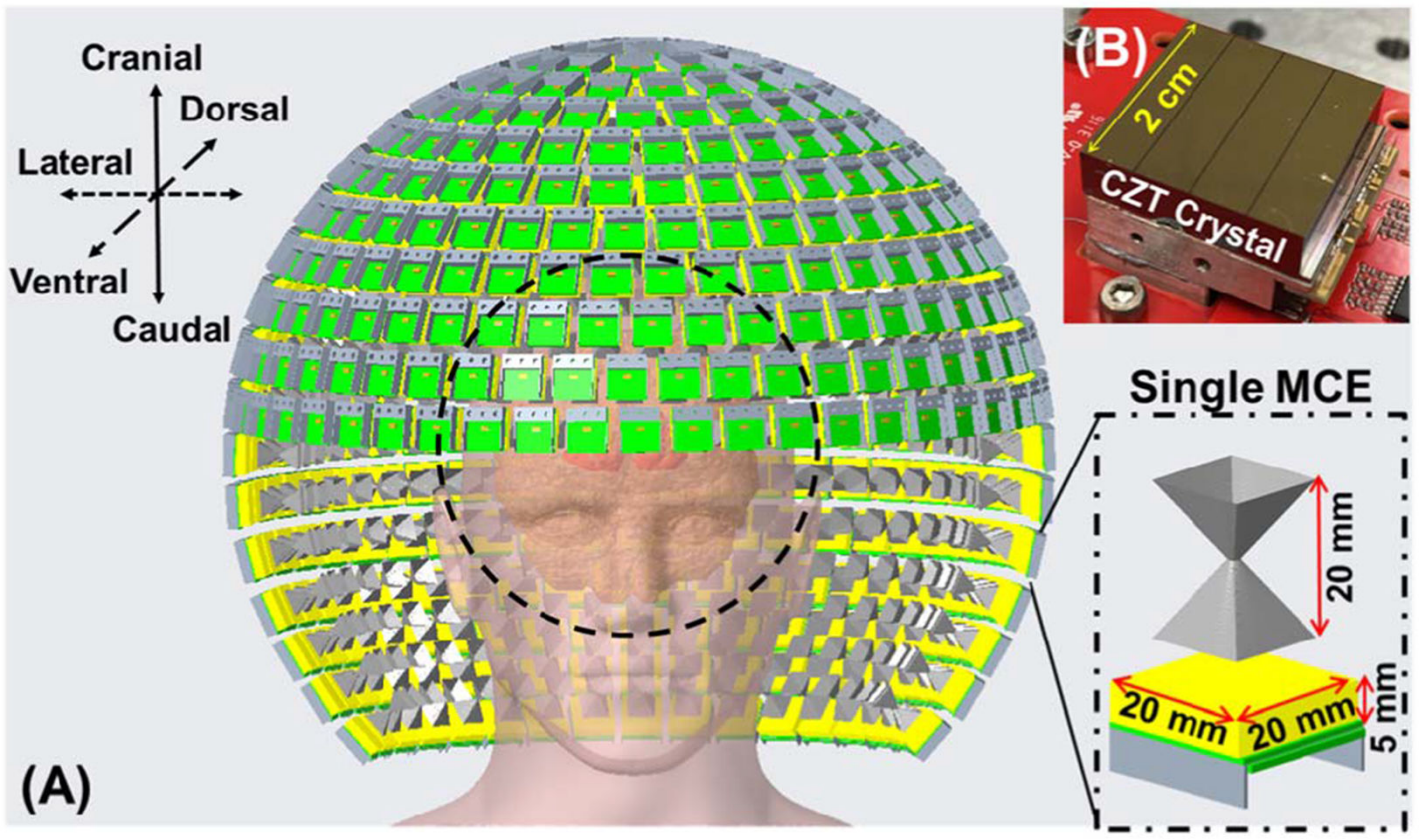
**(A)** 3D drawing of the HelmetSPECT system along with real-sized human head and brain. Only the internal surfaces of the apertures are shown. The dashed circle represents the FOV of 20 cm in diameter. Details of the single MCE are shown in the zoomed section. **(B)** The physical detection unit simulated, based on a CZT/HEXITEC ASIC detector.

The HelmetSPECT system design consists of 502 MCE’s as shown in Fig. 1 and further detailed in Table I in Supplementary Materials. Each MCE consists of a compact CZT detector of 20 mm × 20 mm × 5 mm (Fig. 1B) coupled to a single collimating aperture (Fig. 1 zoomed section). The 502 MCE’s are arranged in 18 rings from top to bottom and located on a spherical surface that has a radius of 196 mm, divided in a hemispherical and neck section. The top hemispherical section has 345 detector modules arranged in 11 full rings and a top cover detector. The neck section has 157 detectors arranged in 7 partial rings. The partial rings cover only 180° for the neck area, leaving an opening in the front (Fig. 1). Each ring accommodates a different number of detectors, as reported in Table I in Supplementary Materials. All the MCE’s are tilted with their main axes pointing towards the center of the FOV in a “spherical arrangement” previously introduced by Ahmed *et al*. in [27] which provides a larger solid angle and, therefore, a better geometrical efficiency than a “multi-ring arrangement”. It is worth to mention that, in case of a PET system such as in [27], the geometric efficiency may increase with the square of the solid angular coverage since a valid event requires the detection of both coincident annihilation photons. In case of the HelmetSPECT system, this relationship is roughly linear. Therefore, an improvement in geometric efficiency by adopting the spherical detector arrangement in a SPECT system is expected, but it will be less effective than in a PET scanner.

In the HelmetSPECT design, we assume that the human brain for most of the population could be contained in a spherical volume-of-interest (VOI) of 20 cm in diameter [31], which is covered by all MCE’s simultaneously. The anatomical directions referred in the current work are shown in Fig. 1. The center of the FOV then coincides with the center of the semi-spherical detection system used in the detector arrangement. This design choice would allow for high-resolution brain imaging in a single stationary acquisition.

### B. Non-Conventional Apertures

In order to deliver a high-performance SPECT system for brain-dedicated nuclear imaging, we have explored a unique system design that combines the SCE gamma camera design with two types of non-conventional apertures, namely micro-slit or micro-ring, and compared these aperture options with traditional loftholes [28].

More details for each proposed aperture are reported in the paragraphs below, and the main specifications are summarized in Table II in Supplementary Materials. The collimator is designed considering SPECT imaging with radiotracers emitting in the 70-250 keV energy range (e.g., Tl-201, Tc-99m, In-111), tungsten is chosen as a material with a total thickness of 2 cm, divided in upper (*t*_*u*_) and lower (*t_l_*) thicknesses, respectively above and below the aperture plane (Fig.3C, D, E).

#### 1) Micro-Slit Collimator:

The use of slits in collimator apertures for SPECT imaging is not a new concept. A SPECT device based on a sliding slit and parallel rake collimator, called Linoview, was reported by Walrand *et al*. in [32], reaching sub-millimeter resolution in whole-body small-animal SPECT imaging. The use of slit-slat, multi slit-slat [33], [34] and skew-slit [35] collimators have been evaluated for preclinical [36], [37] and clinical applications [38], [39]. Metzler *et al*. [40] proposed a multi-pinhole collimator with square openings (0.35 mm × 0.35 mm) for a small-animal SPECT system and carefully investigated its imaging properties and sensitivity [41], [42].

**Fig. 2.**
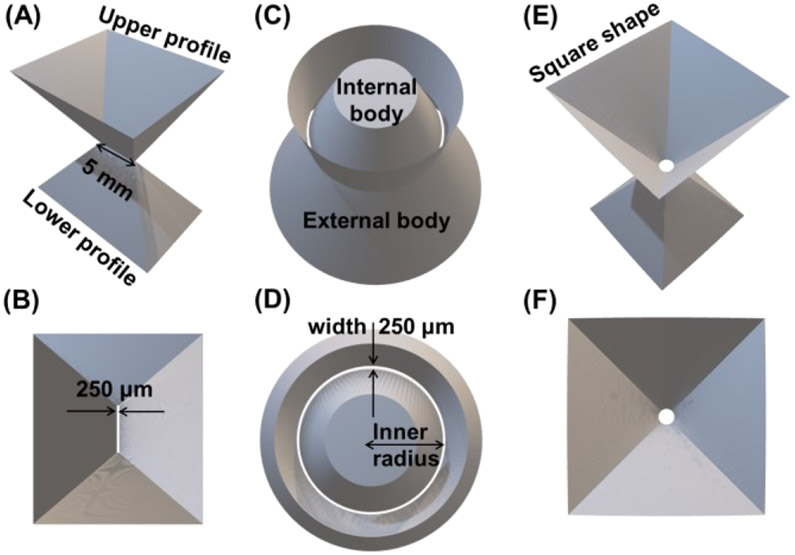
Apertures used in this simulation study: **(A)** single micro-slit insert 250 *μ*m × 5 mm and **(B)** top view; **(C)** single micro-ring insert and **(D)** top view; **(E)** single lofthole insert with 1.5 mm D opening and **(F)** top view.

In this work, we propose and designed two different sets of micro-slit apertures, as summarized in Table II in Supplementary Materials. The micro-slit is a specific type of rectangular pinhole as defined by Xia *et al*. in [41], having the length *l* of the rectangular opening far greater than its width *w* (at least one order of magnitude), with *l* in the millimeter range and *w* in the micrometer range. The first micro-slit (Fig. 2A-B) has an opening of 250 *μ*m × 5 mm (*w* × *l*), and a minification factor (MF) 1:12. The micro-slit *w* is set equal to the intrinsic resolution of the simulated detector and the *l* was chosen to assure proper absorption properties. The second micro-slit has an opening of 150 *μ*m × 6 mm (*w* × *l*), and the same MF 1:12. The micro-slit *w* was chosen as the smallest feature that can be reasonably produced with additive manufacturing techniques for a rectangular opening, whereas the longer *l* compensates for the narrower width.

**Fig. 3.**
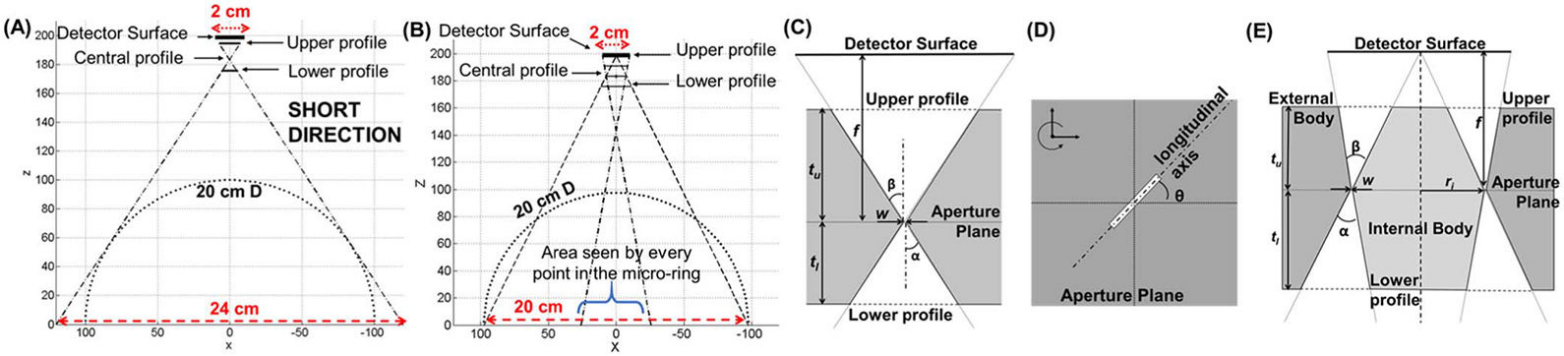
An illustration of the highly de-magnifying geometry used in the SCE gamma camera design. **(A)** Cross-section view of a MCE with a micro-slit aperture, along the short direction. **(B)** Cross-section view of a MCE with a micro-ring aperture. **(C)** Line diagram for the micro-slit aperture along the short direction. **(D)** Micro-slit top view with the main parameters used in the micro-slit rotation. **(E)** Line diagram for the micro-ring aperture.

In the micro-slit design, the narrow opening allows one to collect imaging information at a very high spatial frequency, while the longer dimension helps to maintain a large open fraction of the aperture. The main feature of the micro-slit aperture is that the FOVs along the short and long side of the slit becomes separable [41], offering different spatial resolution properties along the two directions. The walls of the aperture in both directions have different acceptance (*α*) and exit (*β*) angles (Fig.3C): the acceptance angles determine the rectangular FOV covered by the aperture, while the exit angles guarantee that the projection from the micro-slit is properly confined inside the corresponding 20 mm × 20 mm detector module surface. Along the short micro-slit direction, *α* is 27.3°, and *β* is 29.4° for both the micro-slits. Along the long direction *α* is 35.3° and *β* is 21.1° for the 150 *μ*m micro-slit. The 250 *μ*m micro-slit presents the same value for *α* (35.3°), while *β* is 22.6° to compensate for the shorter micro-slit.

Since each individual MCE coupled with a single micro-slit aperture would provide an asymmetrical sampling of the object-space, each micro-slit in the HelmetSPECT design has its longitudinal axis rotated by an angle *θ* about the normal axis of the aperture plane, as shown in Fig.3D. The rotation angles *θ* have values between 0° and 360° in increments of 0.717° ( *θ* = [0 : Δ*θ* : 360° – Δ*θ*] where Δ*θ* = ^360°/^_*N_slit_*_ ≅ 0.717° and *N*_*slit*_ = 502) and are randomly distributed over the HelmetSPECT geometry. For each rotation angle, the squared upper insert profile (close to the detector) is designed to confine the projection within the boundary of the corresponding detector surface, whereas the aperture profile (in the aperture plane) and lower profile (close to the object) are rotated according to the rotation angles *θ*. To avoid possible overlapping of the upper profiles due to the rotation, both the micro-slit apertures have *t_u_* set at 9 mm, compensated by a thicker *t_l_* of 11 mm. Combining >500 micro-slit apertures looking at the object-space across 2/3 of the 4*π* solid angle would help to minimize the impact of the non-isotropic nature of the projection acquired with each individual micro-slit camera.

#### 2) Micro-Ring Collimator:

To the best of our knowledge, we are the first group to propose the use of inserts with ring-shaped openings for SPECT imaging [43]. The micro-ring aperture that we explored in this study has a narrow ring (or annulus) opening and is realized with two parts (Fig. 2C-D and Fig 3E): an external piece with a large circular opening having a radius *r_o_*, and an internal body with a maximum radius *r_i_*, while their difference, *w* = *r*_*o*_ - *r_i_*, defines the width of the ring opening.

These dimensions can be adjusted according to a given imaging application. For example, a narrow width of the ring opening (e.g., 100-500 *μ*m) would lead to a high spatial resolution, while a large ring diameter in the millimeter range (2.5-7.5 mm) can ensure a reasonably large open fraction. The micro-ring aperture design can potentially offer an ultra-high spatial resolution while maintaining a very high sensitivity. In the current simulation study, we used 502 micro-ring apertures with the following specifications (see Table II in Supplementary Materials): an outer radius *r*_*o*_ of 7.5 mm, a ring width *w* of 250 *μ*m, a MF ratio of 1:12, the acceptance angle *α* of 36.3°, and the exit angle *β* of 32.7°. The *β* angles have been designed in order to avoid projection overlapping on the detector surface. To assure proper absorption properties of the internal body, *t*_*u*_ and *t*_*l*_ are 7 mm and 13 mm, respectively. The annulus *w* is set equal to the intrinsic resolution of the simulated detector and the *r*_*i*_ was chosen to assure proper absorption properties of the internal body. The ring-aperture design is illustrated in Fig. 3B and E. Unlike the micro-slit aperture, the micro-ring has a rotationally symmetric sampling such as the lofthole.

#### 3) Multi-Lofthole Collimator:

For comparison, we have also simulated the HelmetSPECT system using two different sets of 502 lofthole apertures, as summarized in Table II in Supplementary Materials. In the current design, the lofthole geometry introduced by K. Deprez and K. Van Audenhaege [12], [28] has been preferred over a traditional pinhole aperture. As known, pinholes have circular-shaped exiting profiles that do not efficiently tile the projections onto the detector surface, resulting in a lower detection efficiency. On the other hand, loftholes offer squared upper (close to the detector) and lower (close to the object) profiles, and the final projections result to be squared for optimal usage of the detector active area.

The first lofthole aperture has 502 identical loftholes of 1 mm diameter and focal distance of 183.23 mm based on a minification geometry with a MF 1:12. The acceptance angle *α* is 27.3°, the exit angle *β* is 29.4°. The open area offered by a single lofthole (0.785 mm^2^) approximately matches the open area offered by the 150 *μ*m × 6 mm micro-slit (0.9 mm^2^) to allow a direct comparison between apertures. We will refer to this collimator as the 1-mm D lofthole aperture.

The option to simulate a lofthole collimator having a diameter of 250 *μ*m (equal to the intrinsic resolution of the simulated detector) has been discarded, since the corresponding geometric system sensitivity would be extremely low, which would defeat the purpose of having 502 detectors around the patient’s head.

The second set of lofthole apertures consists of three different groups of lofthole apertures of different diameters: 168 low-sensitivity-high-resolution loftholes with 500 *μ*m diameter, 167 medium-sensitivity-medium-resolution loftholes with 1.5 mm diameter (Fig.2E and F), and 167 high-sensitivity-low-resolution loftholes with 3 mm diameter. The same focal distance of 183.23 mm and MF 1:12 are used for all the loftholes. Similarly, the same acceptance angle *α* of 27.3°, the exit angle *β* 29.4° are used for all the loftholes. The 500 *μ*m diameter was chosen based on the dimensionality capabilities of rapid additive manufacturing with selective laser melting of tungsten powder. This represents the smallest circular dimension that can be safely produced without evident flaws. The specific combination of the three lofthole diameters is chosen to allow the system to sample the object at high spatial frequencies, while maintaining a relatively high sensitivity. The three types of loftholes are uniformly distributed in the HelmetSPECT geometry: in every ring of the spherical design all three types of lofthole are used, having each lofthole different from the previous and the following in the same ring, as shown in Fig.1S in Supplementary Materials. We will refer to this collimator as the combined 3-type lofthole aperture.

In the spherical arrangement, the centers of each individual MCE were slightly shifted between adjacent rings to ensure the highest number of independent views in the trans-axial direction (Fig. 1S in Supplementary Materials). To compare both lofthole geometries with the micro-slit and micro-ring apertures, the center of each micro-slit and micro-ring is set at the center of each lofthole.

### C. CZT Detectors

The CZT detector used in the design study is based on a compact CZT imaging detector module (Fig. 1B). Each CZT detector crystal has an active area of 20 mm × 20 mm and 5 mm thickness, and is bump-bonded to a HEXITEC ASIC with 80 × 80 pixels of 250 *μ*m ×250 *μ*m pitch [44]. We have designed and developed a compact and scalable external readout electronics [45], which allows users to construct a flexible and reconfigurable detector array. This can be easily tailored for different imaging applications and geometries, including the proposed HelmetSPECT scanner.

The simulated CZT detector carries similar properties as the physical unit described above. Each detector has a detection area of 20 mm × 20 mm divided in a matrix of 80 × 80 pixels of 250 *μ*m × 250 *μ*m in size. In the simulations, the detector was assumed to have a depth-of-interaction (DOI) resolution of 1 mm, which is realized by simulating the detector as five non-overlapping layers of 1 mm in thickness. Each layer of the detector records the projection by accumulating photons reaching the given layer. The DOI sensitivity would reduce the parallax error for photons incident at an oblique angle with respect to the crystal surface, reducing the depth-dependent blurring in the projection. This translates not only in a higher spatial resolution but also in an improved resolution uniformity throughout the FOV. Based on the current design, the narrow open angles of the inserts would confine the direction of the incident photons, but the large minification geometry makes the DOI capability indispensable to achieve a high spatial resolution needed in brain imaging applications. The development of the circuitry for DOI applications is currently underway [46].

### D. System Response Function and Image Reconstruction

To study the performance of the HelmetSPECT system equipped with the different types of apertures, the system response function (SRF) of each design was calculated. The discretized version of the SRF is the system response matrix (SRM) [47] defined as

(1) $$A=\left[\begin{array}{ccc} a_{11} & \cdots & a_{1N}\\ \vdots & \ddots & \vdots \\ a_{M1} & \cdots & a_{MN} \end{array}\right]$$

where each element *a*_*mn*_ gives the probability of a gamma-ray emitted at the *n*^*th*^ source voxel in the object-space and being detected by the *m*^*th*^ detector pixel within a unit imaging time. Each column of the SRM represents the point response function (PRF) for the *n*^*th*^ source voxel in the object-space. The sensitivity value of each voxel *i* in the object-space is then

(2) $$s_{i}=\sum_{j=1}^{M}a_{ij}.$$

Therefore, if ***x*** = [*x*_1_ … *x*_*N*_] denotes the unknown values of the voxels in the object-space, with N the total number of voxels, to estimate the pixel intensities that are underlying the projection data ***y*** = [*y*_1_ … *M_m_*], with M the total number of detector pixels, the following operation is performed:

(3) E[y]=Ax+r

where *r* denotes systematic error components, e.g., the error in the system modeling.

To calculate the SRM, we used a voxel-driven method [47] that we have developed and implemented in our previous studies [19], [29]. Gamma-rays are traced from the center of each voxel in the object-space to the center of each detector pixel through the object volume and the collimator. The SRM includes the attenuation of gamma-rays and detector DOI responses, but it does not model scattered gamma-rays. In general, the HelmetSPECT design is based on CZT/HEXITEC ASIC sensors that offer an excellent energy resolution (FWHM of 1.62 ± 0.26 keV at 122 keV [45]). The superior spectroscopic performance effectively reduces the impact of scattered gamma-rays in the reconstructed images and makes the system well-suited for multi-isotope SPECT imaging.

The SRM is computed, converted and stored in sparse format, using the “sprsin” function in C language [48]. Then, 502 mean projections are calculated by forward-projection according to (3), one for each MCE in the HelmetSPECT scanner. The noisy projections were obtained by adding Poisson noise to the mean values using the “poidev” function in C language [48]. The projections, embedding the DOI information recorded by the 5 layers of the detector, are then used in the 3D ordered subset expectation maximization (OSEM) algorithm [49] with 8 subsets for the image reconstruction. No post-filtering is applied.

### E. Digital Phantoms and Numerical System Performance Measures

#### 1) Fisher Information Matrix:

We computed the Fisher information matrix (FIM) [50] for each of the system-aperture geometries being compared. Given a mapping from the object-space ***x*** to the detection space ***y*** governed by a conditional probability density function *p*(***y*∣*x***), the FIM is defined as

(4) $$J=-E\left\{\left[\frac{\partial \;\log\;p(y|x)}{\partial x}\right]\cdot {\left[\frac{\partial \;\log\;p(y|x)}{\partial x}\right]}^{T}\right\}$$

We computed the FIM for a given system design characterized by the corresponding SRM *A* and a given object *x*. Given the linear Poisson model in (3), the FIM can be written as

(5) $$J=A^{T}\left[\begin{array}{ccc} \frac{1}{{\bar{y}}_{1}} & \cdots & 0\\ \vdots & \ddots & \vdots \\ 0 & \cdots & \frac{1}{{\bar{y}}_{M}} \end{array}\right]A$$

where ${\bar{y}}_{m}$ is the mean projection on the *m*^*th*^ pixel in the detector space. The simulated object *x* has a uniform activity distribution in the entire FOV. To further illustrate the spatial resolution of the given system design, we select the *l*^*th*^ column of the FIM *J*, that is corresponding to a target voxel *l* in the object-space. We then rearrange the elements of ***J**_l_* into a 3D matrix with the same order of the physical voxels in the 3D object-space. The resultant 3D matrix containing the specific column of the FIM is referred to as a FIM image. A FIM image depicts the weighted correlation between the response of the system to gamma-ray emissions at a given target voxel *l* and the response of the system at all other voxels in the object-space, weighted by the variance on the projection data. Examining the FIM image would provide valuable insight into the spatial resolution of an imaging system and the degree of multiplexing in the projection data being introduced by a given system geometry.

#### 2) Digital Phantoms:

To evaluate the performance of the proposed geometries, key performance parameters of sensitivity, spatial resolution, angular sampling, and contrast must be tested. Several digital phantoms having 96 × 96 × 96 voxels of 2 mm × 2 mm × 2 mm in size were used in images reconstructed with noiseless and noisy projections, as described below:

##### a) Defrise phantom:

A noiseless Defrise phantom was simulated and inspected for the presence of artifacts to assess the axial sampling of the system using the different types of collimator apertures. The phantom consists of a 16-cm diameter sphere divided in disks of 6 mm in thickness and spacings. The phantom main axis is placed along the craniocaudal direction of the HelmetSPECT system (Fig.1A).

##### b) Hot-rod phantom:

A hot-rod phantom was used to assess the spatial resolution. The phantom has four groups of hot-rods with diameters ranging from 4 mm to 10 mm and 80 mm in length, placed inside a cylinder with a diameter of 160 mm and axial length of 80 mm. The distance between the center of two neighboring rods is twice their diameter. The resolution phantom was simulated under two different signal-to-background (S/B) ratio conditions: in the first phantom, the hot rods were filled with a solution of 99mTc for a total activity of 1 mCi, while the cylinder was filled with 0.5 mCi in order to have a S/B 20:1. In the second phantom, the hot rods were filled with the same solution of 1 mCi 99mTc, while the cylinder was filled with 2 mCi in order to have a S/B 5:1. Both phantoms are imaged for 30 min and have the main axis placed along the craniocaudal direction of the HelmetSPECT system (Fig.1A).

The *normalized root-mean-square error (NRMSE)* was used to estimate the quantitative accuracy between the reconstructed images and the phantom. The NRMSE was preferred over the RMSE to facilitate the comparison between datasets obtained from the different simulated apertures. The NRMSE is defined as

(6) $$NRMSE=\sqrt{\frac{\sum_{n=1}^{N}(x_{n}-x_{n}^{T}{)}^{2}}{\sum_{n=1}^{N}x_{n}^{T2}}}$$

where *x_n_* is the *n*^*th*^ voxel value in the reconstructed image, $x_{n}^{T}$ the true value of the *n*^*th*^ voxel in the phantom, and *N* is the total number of voxels.

The hot-rod phantom was used also for assessment of the contrast. The *contrast recovery coefficient (CRC)* [12] was calculated for one hot rod of each group (the one closest to the phantom central axis) as

(7) CRC(%)=/(C−1)(μrodj−μbckμbck)×100

where *μ*_*rodj*_ is the mean count in the hot rod *j*, *μ*_*bck*_ is the mean count in the background rod (8 × 8 voxels rod in the center of the phantom) and C the true rod-background ratio from the digital phantom (C =20).

*The contrast-to-noise ratio (CNR)* was calculated as

(8) $$CNR=\frac{|\mu_{rod\;j}-\mu_{bck}|}{\sigma_{bck}}$$

where *σ*_*bck*_ is the standard deviation of the counts in the background rod.

Finally, we computed the *noise coefficient (NC)* as defined by [12]

(9) $$NC(\%)=\frac{\sigma_{bck}}{\mu_{bck}}\times 100$$

and plotted the CRC-NC curves as an additional indicator of the image quality offered by different types of collimators, in terms of image contrast recovery and noise amplification during the iteration reconstruction process. The best collimator should provide the most accurate resolution recovery with a reasonable low noise amplification.

#### 3) Brain Phantoms:

To assess the performance of the system for ictal SPECT applications, two digital brain phantoms were generated: an ictal brain perfusion phantom (Fig.2S(A), Supplementary Materials), and a low-contrast focal lesion detectability phantom (Fig.2S(B), Supplementary Materials). The ictal brain perfusion phantom simulates a realistic ictal brain perfusion for mesial temporal lobe epilepsy (MTLE) according to [51]. It has 16 functional VOIs lateralized in 32 anatomical VOIs, and the normalized uptake ratios (UR), region volumes, and asymmetry indices (AI) are listed in Table III in the Supplementary Materials. The lateralization is needed due to the asymmetric perfusion values between the healthy (right) and ictal (left) hemisphere, typical in MTLE. In the phantom, the Temporal Pole (TPo) region in the ictal hemisphere is the smallest anatomical VOI (circled in Fig.2S(A), first row) with the highest perfusion value due to MTLE (activity concentration of ~ 0.07 *μ*Ci/mL).

The *low-contrast focal lesion detectability phantom* is derived from the ictal perfusion phantom including four spherical lesions with diameters of 5 mm (lesion 1), 6 mm (lesion 2), 7 mm (lesion 3) and 8 mm (lesion 4) located in the TPo (lesion 1), Temporal-Lateral (TL, lesion 2 and 4), Temporal-Mesial (TM, lesion 3) of the ictal hemisphere, as shown in Fig.2S(B). The low lesion-to-background contrast levels were set as 3:2.5:2:1.5:1 for lesion1:lesion2:lesion3:lesion4:background where the background is the white matter region with activity concentration of ~0.056 *μ*Ci/mL. The phantom is used to test the ability of the HelmetSPECT system to detect small low-contrast focal lesions and to delineate their boundaries. More details about the brain phantoms are reported in the Supplementary Materials (Fig.2S). Finally, 2 × 10^9^ photons were simulated in both digital brain phantoms [51], and their orientation follows the anatomical directions shown in (Fig.1A).

In order to examine the potential artifacts solely induced by the collimators used, we firstly reconstructed the images from the ictal perfusion phantom using noiseless projections, with 500 iterations for each collimator simulated. We then produced noisy reconstructions of the focal lesion detectability phantom, with 50 iterations for each collimator simulated. The noisy reconstructions were used to investigate the performance of the HelmetSPECT system under more realistic conditions. The quantitative accuracy of the activity distribution in the reconstructions is evaluated with the numerical measures as detailed below.

The *uptake ratio (UR)* and the *inter-hemispheric asymmetry index (AI)* are two metrics used in SPECT quantification methods [52]. The UR is defined as the ratio between the average counts per voxel measured in a cerebral VOI (*μ_i_*) and the average counts per voxel measured in a reference VOI (*μ*_*Ref*_), chosen as an 8-mm diameter sphere in the healthy (right) cerebellum,

(10) $$UR=\frac{\mu_{i}}{\mu_{ref}}.$$

The *inter-hemispheric asymmetry index (AI)* [51], [52] describes the relative difference between the left and right hemisphere activity with respect to the right hemisphere for each VOI, defined as

(11) $$AI=\frac{\mu_{iL}-\mu_{iR}}{\mu_{iR}}$$

where *μ*_*iL*_ and *μ*_*iR*_ are the mean counts from the VOI *i* in the left and right hemisphere, respectively. This index is needed in applications where the differences between the tracer concentrations in the left and right hemispheres are enhanced, such as in MTLE. The UR’s and the AI’s in all brain VOI’s were calculated and compared to the ones from the phantom. Further details are given in Table III in the Supplementary Materials.

Lastly, for each aperture type and for each lesion in the low-contrast focal lesion detectability phantom, the CRC’s, CNR’s and UR’s were calculated to quantify the accuracy of the reconstruction and the visibility of the focal lesions. For the CRC, defined in (7), C =3 for lesion 1 (5 mm in D), C =2.5 for lesion 2 (6 mm in D), C =2 for lesion 3 (7 mm in D) and C =1.5 for lesion 4 (8 mm in D), while *μ*_*bck*_ is the mean value of the counts in the white matter. For the CNR, defined in (8), *σ*_*bck*_ is the standard deviation of the counts in the white matter. For the UR, defined in (10), the reference region is the white matter.

## III. Results

### A. Spatial Resolution and Sensitivity Tradeoff

**Fig. 4.**
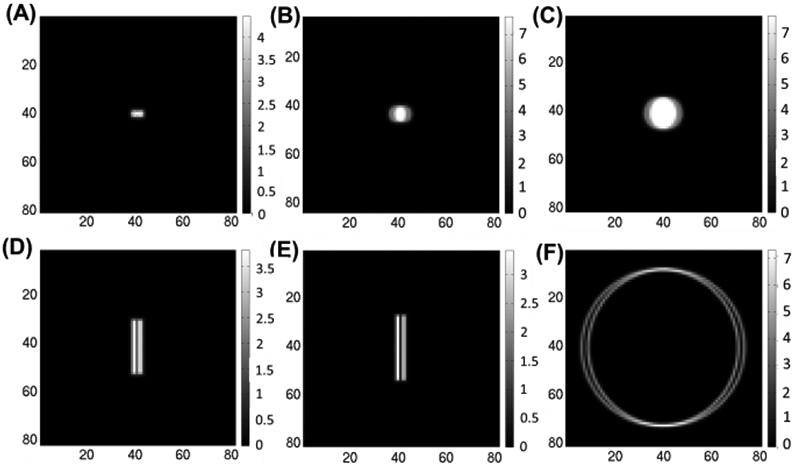
Noiseless projections from two point sources in position [−2,0,0] mm and [2,0,0] mm using **(A)** 500 *μ*m-D lofthole, **(B)** 1.5 mm-D lofthole, **(C)** 3 mm-D lofthole, **(D)** 250 *μ*m × 5 mm micro-slit, **(E)** 150 *μ*m × 6 mm micro-slit, **(F)** micro-ring. The micro-slits in (D) and (E) have the main axis of the slit perpendicular to the direction where the two point-sources are placed.

It is known [53], [54] that to improve the tradeoffs between effective sensitivity and imaging resolution, one needs to minimize the projection overlapping to reduce the ambiguity on the incident direction of the detected photons. To illustrate the tradeoff between spatial resolution and sensitivity offered by the lofthole, micro-slit, and micro-ring apertures, we consider two point-sources located in the FOV and being 4 mm apart from each other as target spatial resolution. In Fig. 4, we compare the simulated mean projections onto the detector module of 20 mm × 20 mm in size through lofthole, micro-slit, and micro-ring apertures. With the lofthole apertures, the projections of the two point-sources are two circular areas largely overlapping (Fig. 4B-C), unless very small loftholes are used (500 *μ*m diameter, Fig. 4A).

In case of the micro-slit aperture (Fig.4D-E), each point source casts a projection in the form of a long straight line, oriented in the same direction as the micro-slit. With the micro-ring aperture (Fig.4F), each point source projection on the detector is a ring of very narrow width. Therefore, the non-conventional apertures codify differently the information in the projections based on two independent parameters: the *l* and *w* for the micro-slit, and the *w* and *r_i_* for the micro-ring. From these illustrations, both micro-slit and micro-ring apertures allowed the projections from the two point-sources to be well separated with none or minimal overlapping. It is worth to notice that the micro-slits in Fig.4D-E have the main axis of the micro-slit perpendicular to the direction where the two point-sources are placed. If the main axis of the micro-slit is parallel to such direction, the projections would be mostly overlapping. In this case, the use of other micro-slits of different orientations within the SCE geometry would assure the acquisition of projections where the two specific point sources are resolved.

**Fig. 5.**
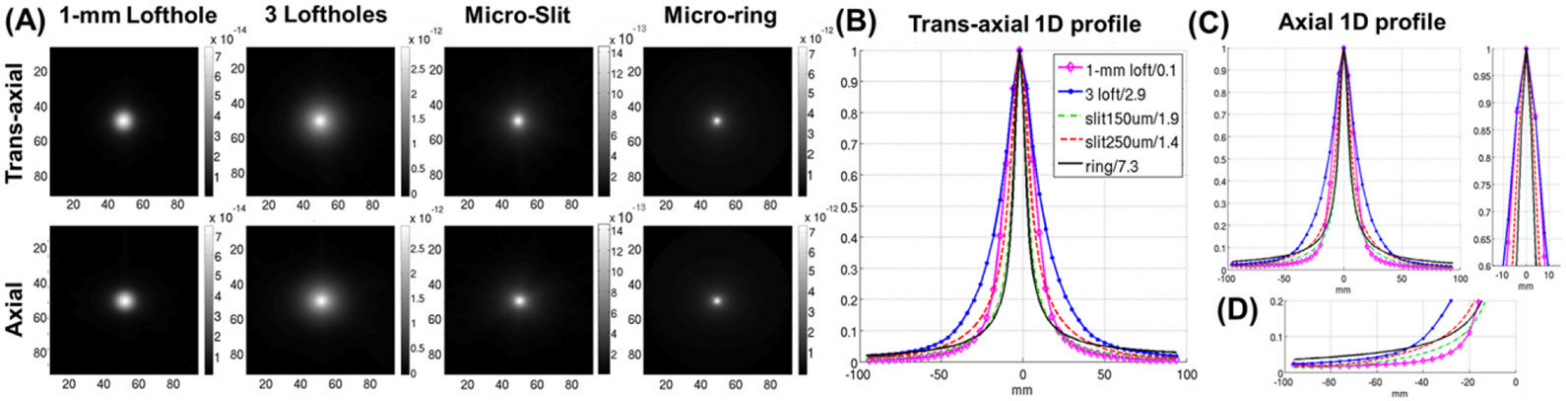
**(A)** Trans-axial **(top row)** and axial **(bottom row)** cross-sections of the FIM column corresponding to the central voxel in the FOV (location [0,0,0] mm) of the HelmetSPECT system coupled with **(first column)** 1-mm D lofthole, **(second column)** 3-types lofthole, **(third column)** 250 *μ*m × 5 mm micro-slit, and **(fourth column)** micro-ring collimator. **(B-C)** Comparison of the 1D profiles, normalized by the peak maximum, along the **(B)** trans-axial and **(C)** axial directions. The values of the peak maxima used in the normalization are reported in the legend for each collimator type.

On the other hand, when two objects are distant, the projections from loftholes and micro-slits do not show overlapping, whereas the micro-ring presents the same feature visible in Fig.4F. This translates in an increased correlation in the micro-ring case between the response of the system to gamma-ray emissions at a given target voxel *l* and the response of the system at all other voxels in the object-space. This is visible on the periphery of the FIM profiles (Fig.5D). The uncertainty in the projection areas is solved using many micro-rings with different orientations within the SCE geometry, that acquire the same information from different sampling angles.

In Fig. 5, we compared the 2D and 1D cross-sections of the FIM images obtained using different apertures. Fig. 5A shows the axial and trans-axial 2-D cross-sections of the central FIM column corresponding to a target voxel centered at [0,0,0] mm, which demonstrated an isotropic performance resultant from the use of all the apertures. The spreading of the FIM image around the target voxel is narrower in case of micro-slit and micro-ring apertures (16 mm FWHM for micro-slit of 250 *μ*m width, 12 mm FWHM for the micro-slit of 150 *μ*m width and micro-ring), while clearly wider for loftholes (20 mm FWHM for the 1-mm D lofthole, 26 mm FWHM in the SCE aperture combining 3 types of loft holes of different diameters). The comparison was also illustrated with the 1-D cross-sections in the axial and trans-axial direction as shown in Fig. 5B-D. These results suggest that the use of micro-ring and micro-slit apertures leads to a reduced correlation between the system responses to a target voxel and to its adjacent voxels, which would imply a better spatial resolution. At the same time, the peak value in the FIM image for the HelmetSPECT system with micro-ring aperture is 2.5 times higher than the corresponding peak value using the combined 3-type lofthole collimator, which implies that the micro-ring apertures also allows for a much higher peak sensitivity.

Lastly, we verified that the combination of 502 micro-slits rotated by angles *θ* about the normal axis of the aperture plane minimizes the impact of the asymmetrical sampling provided by each single micro-slit. This is illustrated by the FIM images corresponding to 18 voxels within the FOV, located at 2.5 cm, 5 cm and 7.5 cm away from the center, along the ventro-dorsal, cranio-caudal, and lateral directions, as shown in Fig.3S(A) in the Supplementary Materials. The FIM images corresponding to the target voxel are nearly isotropic in the coronal, horizontal and sagittal planes as shown in Fig. 3S(B-C). By comparison, the corresponding FIM images obtained by using the 1-mm D lofthole are also shown in Fig. 3S(B-C). This indicates that the use of the large number of micro-slit apertures would lead to an isotropic imaging property. Further studies are planned to determine the optimum number of micro-slits needed to satisfy this condition.

### B. Gamma-Ray Penetration

The use of the micro-slit and micro-ring apertures offers another practical benefit in terms of edge penetration [55]. Due to the knife-edge geometry commonly used in lofthole apertures, the thickness of the high-density collimator material (e.g., tungsten or lead) is strongly reduced close to the opening, which leads to appreciable probability for gamma-rays to penetrate the collimator volume and then reach the detector surface. This effect results in degradation in image quality, such as loss in spatial resolution. In the micro-slit geometry, the edge penetration becomes a one-dimensional problem rather than two-dimensional: the collimator material is thin close to the narrow width of the micro-slit, while it is thick in the perpendicular direction, assuring a proper absorption for oblique gamma-rays. Similarly, the micro-ring insert assures an increased material thickness both in the external piece and in the internal body.

To allow the comparison in terms of degree of aperture penetration among the different geometries proposed, we refer to the gamma-rays that go through the designated open area without attenuation as “signal photons”, and the gamma-rays that should be stopped by the collimator but instead penetrate through the aperture material and reach the detector as “noise photons”. Due to the voxel-driven method used to estimate the SRM, we were able to determine the attenuation path of each tracing ray (from every given image voxel to every given detector pixel) that passes through the aperture and to estimate the corresponding attenuation

(12) ∕I0I=e−μwx

where *I*_*o*_ is the ray intensity entering the collimator, *I* is the ray intensity exiting the collimator, *μ*_*w*_ is the attenuation factor of tungsten at 140 keV and *x* is the attenuation path.

We then define the “aperture signal-to-noise ratio” (aperture SNR) as:

(13) apertureSNR=∑signalphotons(∕IoI=1)∑e−μwx∗noisephotons(∕IoI<1)

being the ratio of the signal photons detected and the noise photons penetrated, weighted by the corresponding attenuation through the collimator material (equal to 1 for the signal photons). The “aperture SNR” is a parameter meant to quantify the degree of aperture penetration with different types of apertures.

Under the condition of a uniform source distribution in the entire FOV, Fig.4S(B) in the Supplementary Materials reports the aperture SNR values for the different apertures proposed: the lofthole with 0.5 mm D showed an aperture SNR of 120.5, the lofthole with 1 mm D 274.2, the lofthole with 1.5 mm D 352.2, the lofthole with 3-mm D 420.3, whereas the aperture SNRs from the non-conventional apertures are: 316.5 for the 150 *μ*m micro-slit, 386.4 for the 250 *μ*m micro-slit, and 438.5 for the micro-ring.

These results demonstrate that for conventional apertures offering comparable or larger opening areas (e.g.,1.25 mm^2^ for the 250 *μ*m micro-slit, comparable to a 1.5 mm diameter lofthole (~1.76 mm^2^), 0.9 mm^2^ for the 150 *μ*m micro-slit comparable to a 1 mm diameter lofthole (~0.785 mm^2^), 5.69 mm^2^ for the micro-ring and 7.07 mm^2^ for the 3-mm D lofthole), the non-conventional apertures offer better penetration properties while providing narrow openings in the micrometer range to allow for SPECT imaging at a very high spatial resolution.

### C. System Sensitivity

**Fig. 6.**
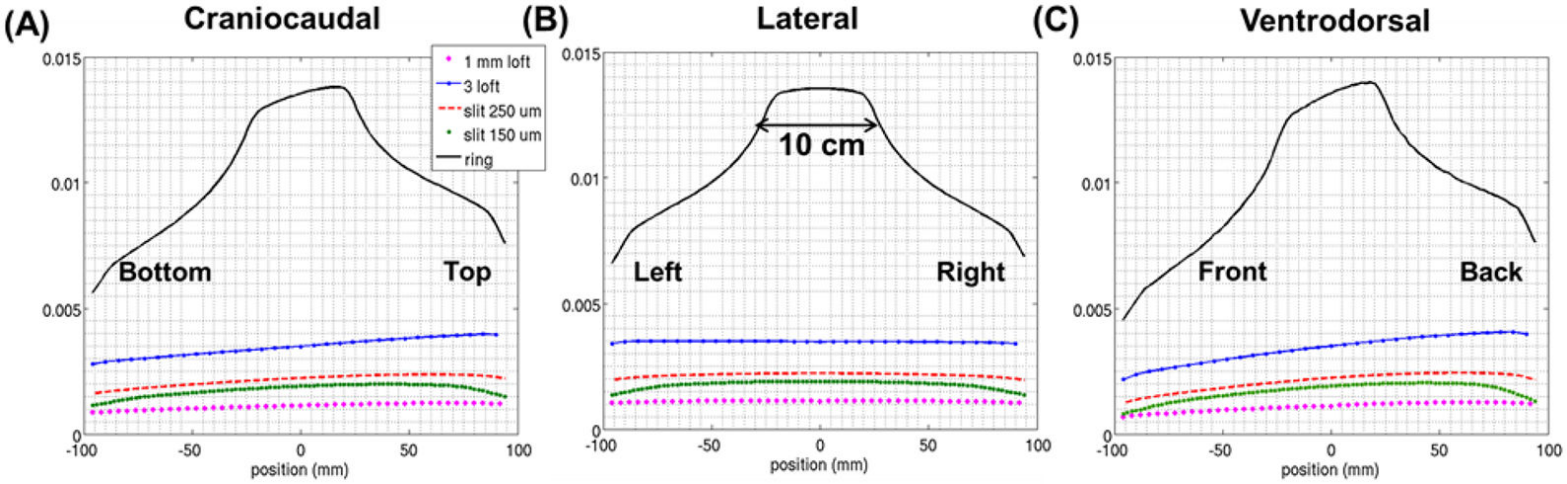
HelmetSPECT sensitivity 1D profiles along the **(A)** craniocaudal, **(B)** lateral, and **(C)** ventrodorsal direction using the different simulated apertures.

The global sensitivity of the HelmetSPECT system is estimated according (2) and the sensitivity profiles are shown in Fig. 6. In the calculation, we considered the material efficiency of 5-mm-thick CZT crystals at 140 keV, having a linear attenuation of 0.354 mm^−1^ at 140 keV, and therefore a probability of interaction of ~83% [56]. As visible, no valleys of sensitivity appear since all the apertures are designed to correctly focus on the FOV, and central sensitivities range from 0.11% (for the 1-mm D lofthole) to 1.38% (for the micro-ring). However, the system sensitivity is affected by the missing detectors from the front opening in the HelmetSPECT geometry (Fig. 6C). As expected, the sensitivity profiles from the 1-mm D lofthole and 150 *μ*m micro-slit collimator show comparable values (0.11% and 0.19%, respectively). While the lofthole and micro-slit apertures provide a uniform sensitivity across the FOV, the micro-ring aperture shows a sensitivity consistently higher than 1% across a central area of 10 cm in diameter since the central portion of the FOV is seen by all the points in the micro-ring aperture (Fig. 3B).

### D. Phantom Studies

Considering the different performance of the HelmetSPECT system with the five different collimators, the convergence rates of the reconstruction would be different for each case. The number of iterations in the OSEM reconstruction used for the subsequent qualitative and quantitative assessment was chosen as follows. The NRMSE over the entire volume was calculated for each iteration. If the NRMSE reaches a minimum (as in the case of reconstructions with noisy projections), then we choose the image at the iteration corresponding to the minimum. In the case of reconstruction with noiseless projections, the NRMSE decreases asymptotically, and we choose the iteration where the difference in the NRMSE between two adjacent iterations is less than 0.1 %_*o*_.

For all images presented in this paper, the noiseless reconstructions are unfiltered and displayed with a slice thickness of 2 mm. The noisy reconstructions from the brain phantoms are filtered with a 3D 6 mm-FWHM Gaussian filter and displayed with a slice thickness of 2 mm. The images from brain phantoms are shown according the radiological display convention.

#### 1) Defrise Phantom:

**Fig. 7.**
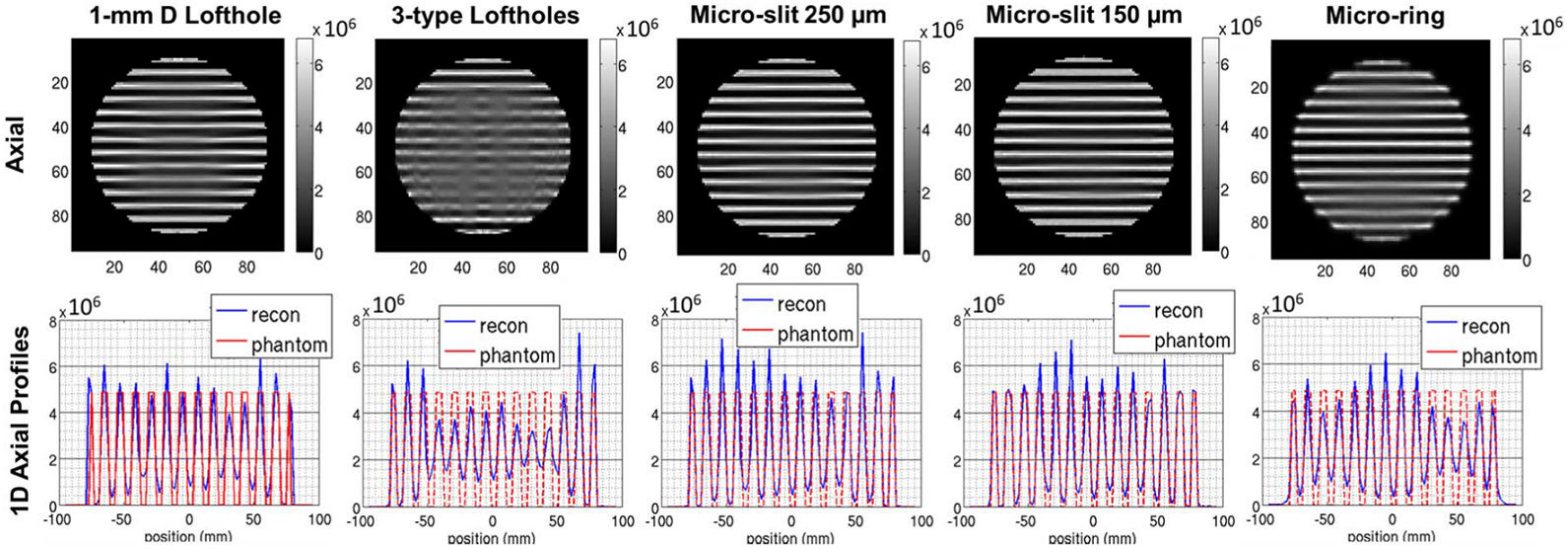
Defrise phantom with 6-mm-thick disks and spacings: **(first row)** noiseless reconstructed images, **(second row)** 1D axial profiles at the corresponding OSEM stopping iteration using: **(first column)** 1-mm D lofthole (1701^st^ iteration), **(second column)** 3-types lofthole (2476^th^ iteration), **(third column)** micro-slit 250 *μ*m × 5 mm (627^th^ iteration), **(fourth column)** micro-slit 150 *μ*m × 6 mm (331^st^ iteration), **(fifth column)** micro-ring (815^th^ iteration). Same colorbar is used, images are unfiltered with 2-mm slice thickness. The main axis of the phantom is placed along the craniocaudal direction of the HelmetSPECT system.

Fig. 7 shows the images reconstructed with noiseless projections. The axial sampling is assessed by plotting the central axial profile across the phantom, which is shown in the second row in Fig. 7. The individual disks of the Defrise phantom are well resolved in the case of 1-mm D lofthole, 250 *μ*m-width micro-slit, 150 *μ*m-width micro-slit, and micro-ring, which indicates that axial sampling completeness is achieved. In the case of micro-ring aperture (5^th^ column in Fig.7), it is noticeable a blurring in the top 4 disks and bottom 5, while it disappears for the central 5. This is due to the increased sensitivity and angular sampling in the central FOV region (see Fig. 6, black line). For the collimator with 3 types of loftholes of different diameters, the angular sampling provided by each type of lofthole is sparser than the case of 1-mm D lofthole collimator. Additionally, only the two smallest loftholes could allow to resolve the disk features at the center of the FOV. The reduction of the number of loftholes able to visualize the feature and the sparser angular sampling led to the degraded resolution in the central area of the FOV, so that the disk features are not well-resolved.

#### 2) Hot-Rods Phantoms:

**Fig. 8.**
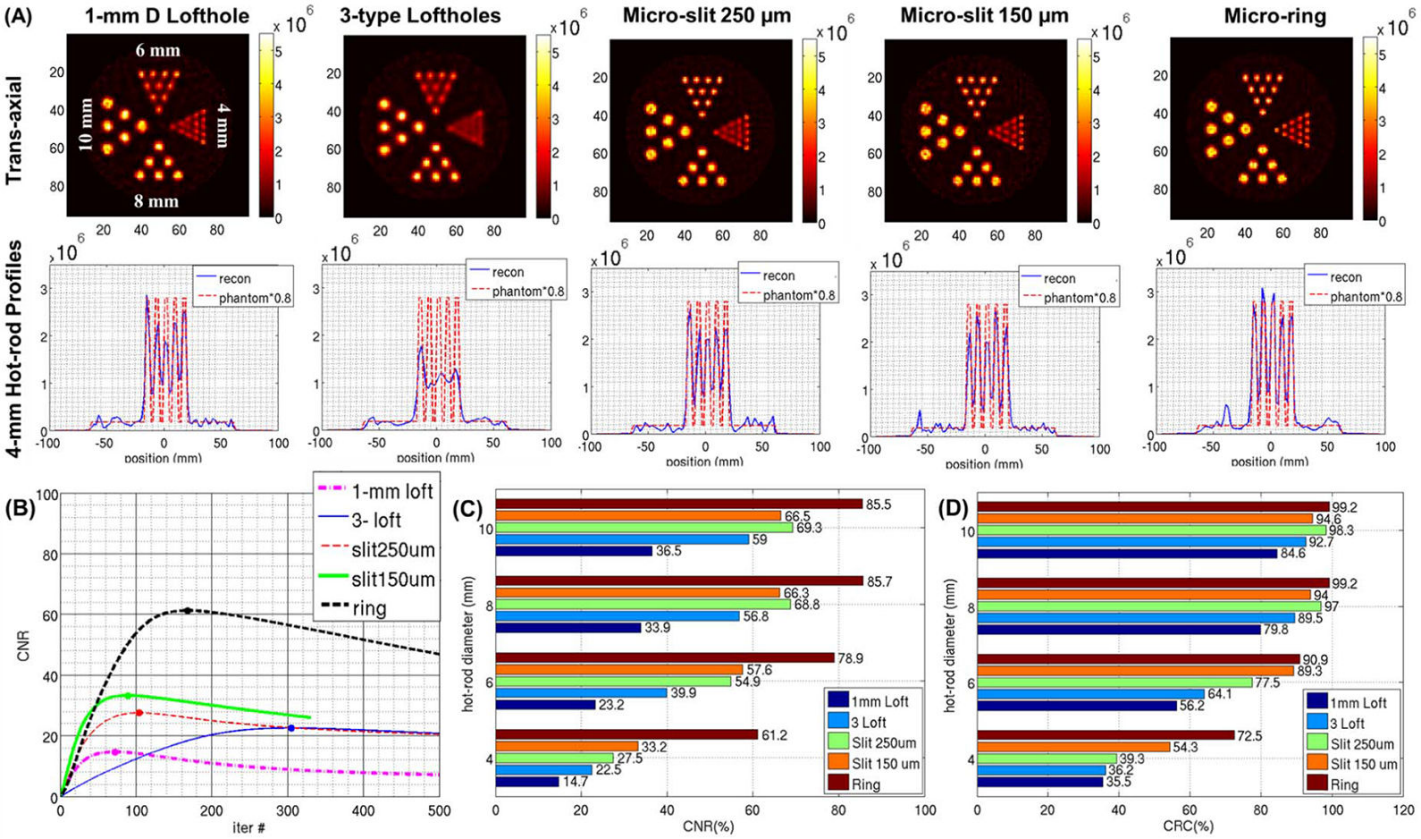
Hot-rod phantom with S/B 20:1: **(A) (first row)** trans-axial view of the noisy reconstructed images, **(second row)** profiles of the 4-mm hot-rods at the corresponding OSEM stopping iteration using: **(first column)** 1-mm D lofthole (247^th^ iteration), **(second column)** 3-types lofthole (381^st^ iteration), **(third column)** micro-slit 250 *μ*m × 5 mm (255^th^ iteration), **(fourth column)** micro-slit 150 *μ*m × 6 mm (173^rd^ iteration), **(fifth column)** micro-ring (385^th^ iteration). Same colorbar is used, images are unfiltered with 8-mm slice thickness (average over 4 central slices) for the trans-axial view. The main axis of the phantom is placed along the craniocaudal direction of the HelmetSPECT system. The profile from the phantom was multiplied by 0.8 to allow comparison. **(B)** CNR curves over iteration for the 4-mm D hot rod. The maximum value and corresponding iteration is shown with a dot. **(C)** Maximum CNR’s across the different hot-rod diameters and apertures; **(D)** CRC’s across the different hot-rod diameters and apertures at the iteration with the maximum CNR.

**Fig. 9.**
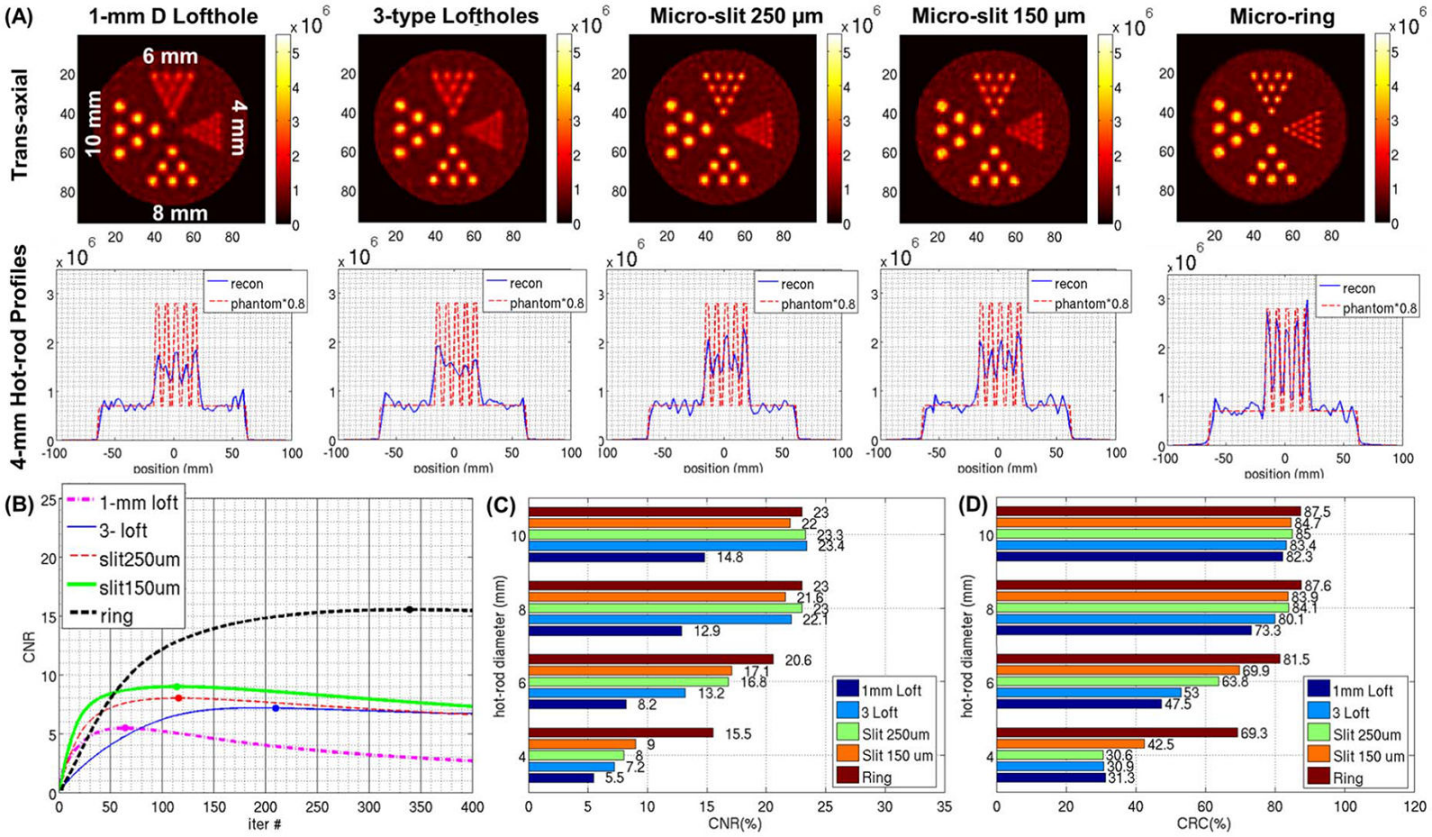
Hot-rod phantom with S/B 5:1: **(A) (first row)** trans-axial view of the noisy reconstructed images, **(second row)** profiles of the 4-mm hot-rods at the corresponding OSEM stopping iteration using: **(first column)** 1-mm D lofthole (126^th^ iteration), **(second column)** 3-types lofthole (241^st^ iteration), **(third column)** micro-slit 250 *μ*m × 5 mm (166^th^ iteration), **(fourth column)** micro-slit 150 *μ*m × 6 mm (122^nd^ iteration), **(fifth column)** micro-ring (276^th^ iteration). Same colorbar is used, images are unfiltered with 8-mm slice thickness (average over 4 central slices) for the trans-axial view. The main axis of the phantom is placed along the craniocaudal direction of the HelmetSPECT system (Fig. 1A). The profile from the phantom was multiplied by 0.8 to allow comparison. **(B)** CNR curves over iteration for the 4-mm D hot rod. The maximum value and corresponding iteration is shown with a dot. **(C)** Maximum CNR’s across the different hot-rod diameters and apertures; **(D)** CRC’s across the different hot-rod diameters and apertures at the iteration with the maximum CNR.

Figs. 8A and 9A show the noisy reconstructions from the hot-rod phantom with a S/B ratio of 20:1 and from the hot-rod phantom with a S/B ratio of 5:1, respectively. In both phantoms, the 4 unfiltered central adjacent slices were averaged together in the trans-axial section to suppress small local fluctuations due to noise. From visual inspection of the resulting images in Fig.8A, with the 1-mm D lofthole aperture, and with both the micro-slit and micro-ring apertures, the smallest hot-rod group that can be resolved is the 4-mm group. With the aperture having 3 types of loftholes of different diameters, the smallest hot rods that can be resolved is the 6-mm diameter. Under higher noise conditions, as shown in Fig.9, the micro-ring aperture shows a clear advantage in resolution, where the 4-mm hot rods are well resolved, while all the other collimators can only resolve the hot rods of 6-mm diameter. The 1-D cross-sections through the top row of the 4-mm hot rods are shown in the second row of Fig. 8A and 9A. To evaluate the ability of the HelmetSPECT system to reproduce the contrast in the object, we compute the CNR and CRC values, defined in (7) and (8), for hot rods of all diameters across the 50 noisy iterations for both resolution phantoms. As shown in Fig. 9B, for each aperture, we choose to use the image reconstructed with the number of iterations leading to the maximum CNR to compute both the CNR and CRC values. The corresponding CNR and CRC values are reported in Fig. 8C-D and 9C-D. This indicates the accuracy of the reconstruction at the best hot-rod detectability condition. As we expected, the CRC and CNR values decrease with decreasing hot-rod diameter for all the apertures used in both phantoms, showing similar trends under different background conditions. The micro-ring consistently offers the best performance for all the hot-rot diameters and for both figures-of-merit, while the 1-mm D lofthole performs the worst, due to the lowest system sensitivity. The CNR values in the S/B 5:1 phantom are lower than the ones in the S/B 20:1 case (Fig. 8C-9C), and the performance between non-conventional micro-slit and micro-ring collimators and combined 3-type lofthole collimator are comparable for the two largest hot-rods in the S/B 5:1 case (Fig.9C). In the CRC results (Fig. 8D-9D), while all apertures perform well for the larger 8-mm and 10-mm hot-rods, their performance varies substantially for the smaller 6-mm and 4-mm hot-rods where the micro-ring significantly outperforms the other collimators. For further comparison of the image quality offered by different types of aperture, we use the same dataset of Fig.9 to plot the CRC-NC curves as shown in Fig. 5S in the Supplementary Materials. Fig. 5S contains a total of 20 curves that are corresponding to the five aperture options and the four hot-rods of different diameters for the full range of iterations. In this comparison, no aperture provides the complete recovery of the rod contrast (i.e., CRC of 100%). Nevertheless, the micro-ring consistently offers the best contrast recovery with the lowest noise conditions for all four rod diameters. Whereas the performance of the loftholes and micro-slits are comparable for the hot rods with the biggest (10 mm) and the smallest diameter (4 mm), micro-slits outperform loftholes in the 6 mm and 8 mm case. As expected, the collimators that have lower sensitivity (Fig.6), namely the 150 *μ*m micro-slit and 1-mm D lofthole lead to reconstructed images with amplified noise.

#### 3) Brain Phantom:

**Fig. 10.**
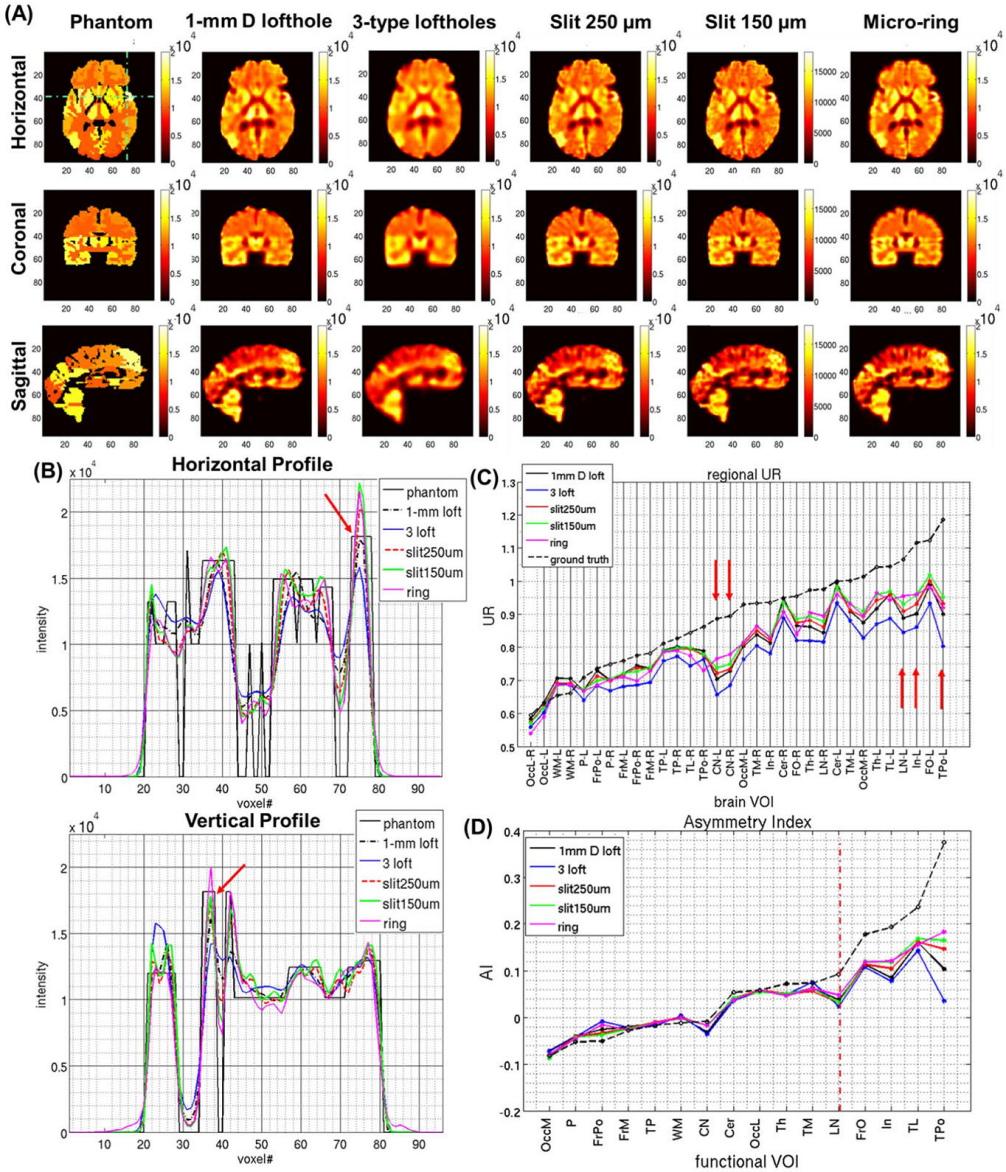
**(A)** Brain ictal perfusion phantom: **(first row)** horizontal, **(second row)** coronal, **(third row)** sagittal central plane from the noise-less reconstructed images at the corresponding OSEM stopping iteration: **(first column)** digital phantom, **(second column)**1-mm D lofthole (167^th^ iteration), **(third column)**3-types lofthole (246^th^ iteration), **(fourth column)** micro-slit 250 *μ*m × 5 mm (217^th^ iteration), **(fifth column)** micro-slit 150 *μ*m × 6 mm (168^th^ iteration), **(sixth column)** micro-ring (386^th^ iteration). Same colorbar is used, images are unfiltered with 2-mm slice thickness. The images are shown according to the radiological display convention. **(B)** Horizontal and vertical 1D profiles through the most interesting feature of the phantom (green dashed lines in A), i.e. the lesion in the left temporal lobe, pointed by the red arrows. **(C)** UR’s for the 32 anatomical brain VOI’s across the different apertures simulated, ordered with increasing perfusion value (black dashed line). **(D)** AI’s for the 16 functional brain VOI’s across the different apertures simulated, ordered with increasing AI value (black dashed line).

Fig. 10A shows the central coronal, sagittal and horizontal cross-sections from the ictal brain perfusion images reconstructed using noiseless data. In these results, the brain perfusion patterns are well preserved with no obvious distortions or artifacts for all the apertures simulated, along with the perfusion asymmetry between the two hemispheres. While the 1-mm D lofthole collimator shows a similar performance to the 250 *μ*m micro-slit, the combination of 3 types of loftholes provides the most blurred images, which is due to the use of large diameter loftholes in the aperture. The images that best reproduce the true perfusion pattern are the ones offered by the micro-rings and 150-*μ*m micro-slits. The 1D profiles through the lesion in the left temporal lobe are shown in Fig. 10B (marked with red arrows). The different resolution performance of the simulated collimators strongly affects the reconstructed images shown in Fig.10A. The discrepancies between the reconstructed images and the phantom are the results of partial volume effects [57], where the image reconstruction is affected by the complex 3D geometry and the heterogeneous perfusion patterns in previous and next slices of the brain phantom.

Fig. 10C shows the UR values estimated from the unfiltered noiseless reconstructions (shown in Fig. 10A) for the 32 anatomical brain VOIs (ordered with increasing perfusion value, black dashed line). Fig. 10D shows the inter-hemispheric AI values for the 16 functional brain VOIs (ordered with increasing AI, black dashed line). The arrows in Fig. 10C point at the regions with the biggest difference between the reconstructed UR values and the ground truth, namely caudate nuclei (CN), lenticular nuclei (LN), insula (In) and left TPo. The reasons why the estimation in these areas is poorer are considered as the following: CN, LN and In regions are small and internal brain regions (Fig. 2S(A)), whose reconstruction is likely to be affected by the partial volume effects from surrounding areas. The TPo region in the left (ictal) hemisphere is the smallest anatomical VOI with the highest perfusion value due to MTLE, located peripherally in the hemisphere. These results consistently show that the combination of 3 types loftholes in the aperture produces the worst results, while the other four collimators have comparable performances, with the non-conventional micro-slit and micro-ring apertures offering better estimation for higher UR’s (UR ≥0.9). In Fig. 10D, it is evident that the five collimators have similar performance in terms of the accuracy in reproducing the AI values in regions with the true AI values ≤0.09. For regions with higher true AI values, e.g., AI ≥0.2, the micro-slit and micro-ring apertures offer more accurate estimations than the system equipped with loftholes.

**Fig. 11.**
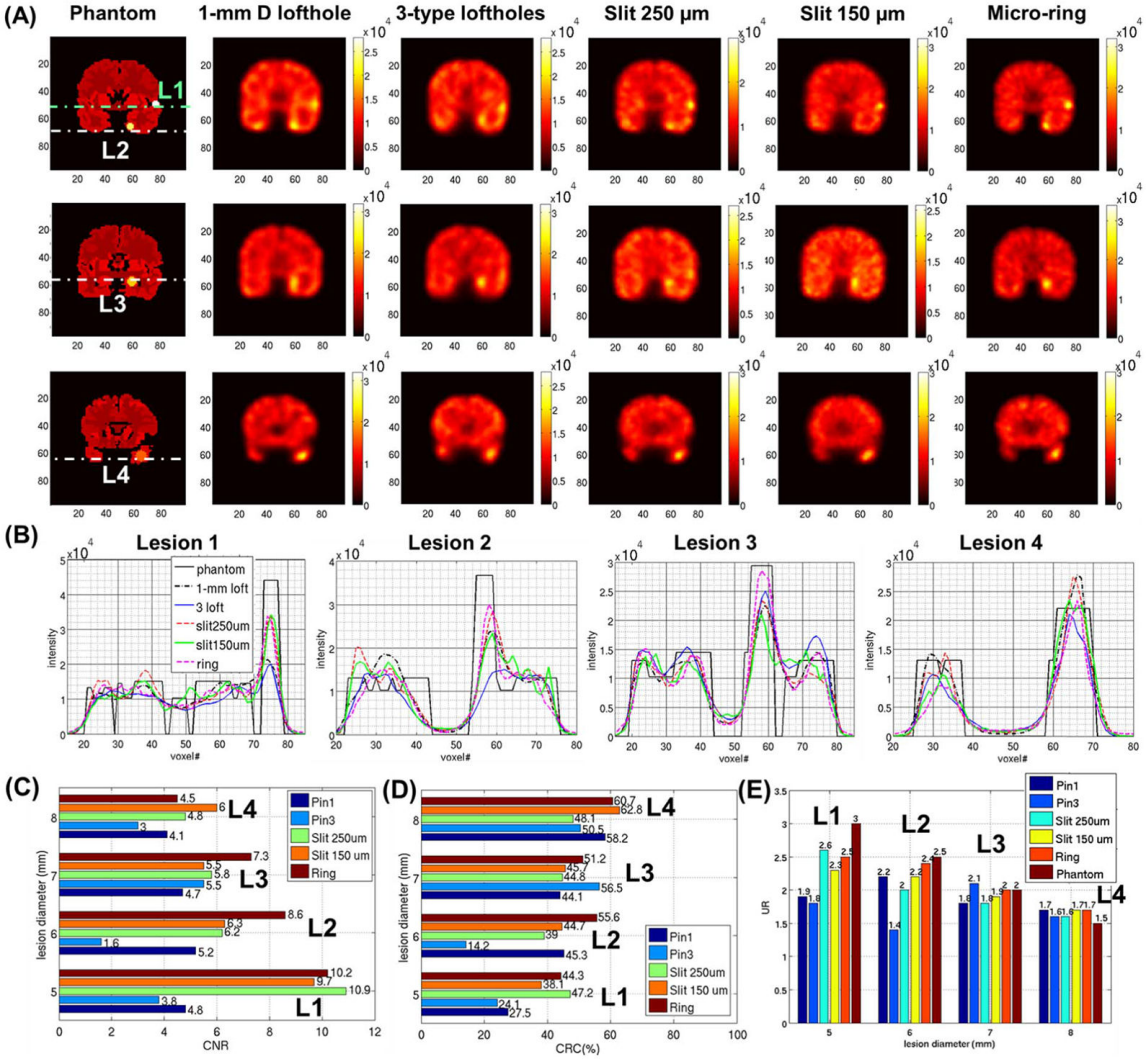
**(A)** Low-contrast focal lesion detectability brain phantom at the iteration with lowest NRMSE, coronal plane: **(first column)** digital phantom, and noisy reconstructions using **(second column)** 1-mm D lofthole (10^th^ iteration), **(third column)** 3-type lofthole (21^st^ iteration), **(fourth column)** micro-slit 250 *μ*m × 5 mm (8^th^ iteration), **(fifth column)** micro-slit 150 *μ*m × 6 mm (5^th^ iteration), **(sixth column)** micro-ring (27^th^ iteration). Images are filtered with a 3D 6-mm FWHM Gaussian filter and have 2-mm slice thickness. The images are shown according to the radiological display convention. **(B)** Horizontal and vertical unfiltered 1D profiles of the four lesions. **(C)** CNR, **(D)** CRC and **(E)** UR values from the lesions across the different apertures simulated.

Fig. 11A shows the coronal slices from images of the low-contrast focal lesion detectability phantom reconstructed with noisy projection data. We also provide the subtraction images in Fig. 6S in the Supplementary Materials to better visualize the low-contrast lesions. These subtraction images are obtained by normalizing and subtracting the reconstructed images of the non-lateralized perfusion phantom without lesions from the reconstructed images of the low-contrast focal lesion detectability phantom. Fig. 11B shows the unfiltered 1D profiles of the four low-contrast focal lesions, which displays the performances offered by different types of collimators in following the variations of activity concentration in the reconstructed images. Finally, Fig. 11C-E show the quantitative accuracy of the 3D reconstructions in terms of UR’s, CRC’s and CNR’s of the four low-contrast lesions from the different apertures simulated. The values reported in Fig. 11C-E correspond to the iteration having the highest CNR value in the reconstruction process.

## IV. Discussion

We present the design of a dedicated-brain SPECT scanner, the HelmetSPECT system, that combines ultrahigh resolution CZT imaging detectors, the SCE gamma camera design, and micro-slit and micro-ring apertures, to deliver a clinical brain scanner with a very high spatial resolution and an excellent sensitivity over a clinically relevant FOV.

As we have demonstrated in [19], [29] and in this paper, this unique system design offers several attractive aspects for SPECT imaging as summarized below. *First,* the use of many MCE’s (~500) collecting photons from a large number of angles around the object, leads to an excellent imaging resolution and a very high sensitivity over a large FOV of 20 cm diameter. *Second,* the SCE design with the vast number of degrees-of-freedom in the geometrical parameter space allows a great flexibility for tailoring the imaging system to a given imaging application. Note that the combination of different types of MCE’s and/or the choice of the best design parameters (such as micro-slit and micro-ring dimensions) would require an exhaustive optimization process going through an infinitely large number of design options. This is out of the scope of the current work. The design parameters presented have not been individually optimized, but have been chosen to satisfy sensitivity, spatial resolution, FOV and proper absorption requirements. We plan to address this topic in future studies. *Third,* the SCE camera design leads to a very compact detection system (as shown in Fig. 1). This would allow the HelmetSPECT system to be easily moved in and out and operated in surgical operating rooms, intensive care units, and potentially be used at the bed side. *Fourth,* the highly de-magnifying geometry leads to a reduced CZT detector volume in comparison to magnifying geometries and therefore a lowered hardware cost, which may be a trivial benefit from research standpoint, but is critically important for future widespread deployment of high-performance CZT-based clinical SPECT systems. Currently, the limited spatial resolution typical of scintillation detectors is less likely to allow for such minifying geometry. In case of scintillators, the adoption of a magnifying geometry would require a larger detection area and many apertures would need to be suppressed to avoid multiplexing effects, reducing the overall system angular sampling and sensitivity. As an additional consideration, it is worth pointing out that the total detection area of 2008 cm^2^ is similar to the area of a single camera head (39 cm × 51 cm) in the dual-head GE Discovery NM/CT 670 CZT scanner [58]. Future studies will explore the possibility to reduce the total number of detector modules needed, for example introducing a checkerboard pattern. *Finally*, since in each MCE the detector module is coupled with only one collimator aperture, the SCE geometry does not introduce projection overlapping. This prevents the multiplexing of projections, which is known to degrade the image quality for a modest gain in detection efficiency [53], [54].

Based on the results in Figs. 7-11 and 5S, we can conclude that: (*a*) the system designs based on non-conventional micro-slit and micro-ring apertures could lead to an improved spatial resolution and sensitivity compared to the loftholes apertures, which is translated into improved CRC, CNR and reduced noise amplification. (*b*) The specific performance improvement of the micro-slit and micro-ring apertures depend on the imaging task, which is more significant for reconstructions at a higher imaging resolution and for quantification of small lesions, such as the 4-mm diameter hot rods or the small lesions in the low-contrast focal lesion detectability phantom, and less significant for imaging at a lower spatial resolution and for quantification of larger lesions, such as the 10-mm diameter hot rods or bigger lesions. (*c*) The combination of 3 types of loftholes having different diameters improves the system sensitivity but offers the lowest spatial resolution in this study.

While a uniform sensitivity in the FOV is usually preferred, the sensitivity obtained from the micro-ring aperture shows a central area of 10 cm in diameter having values consistently higher than 1%. This would be useful in SPECT imaging applications where brain VOIs have well-known locations with activity distributions centrally concentrated, such as in dopamine receptor imaging where the tracer uptake is concentrated in the striatum [59]. As we have shown in Figs. 8-9 and 5S, the micro-ring aperture results in the highest spatial resolution (<4 mm spatial resolution in both S/B conditions) and at the same time, it provides a significantly improved quantitative accuracy, as evident by the highest CRC and CNR for quantifying the tracer uptakes in the hot-rods of all diameters. It is evident that the improvement in CNR is substantial for smaller features where high spatial resolution and high sensitivity are required, while it is limited for bigger features. In the case of the smallest features (4-mm and 6-mm D), the higher spatial resolution offered by micro-ring and micro-slits reduces the partial volume effects from surrounding areas and allows to correctly estimate the contrast. On the other hand, the biggest features (8-mm and 10-mm) do not require such high spatial resolution and, therefore, are reconstructed embedding higher noise levels. This causes an increase in the standard deviation in (8) and a reduction of the CNR.

The performance benefits from using the micro-slit and micro-ring apertures are further demonstrated by the qualitative and quantitative comparison of the noiseless images of the brain ictal perfusion phantom (Fig. 10), where an improved image reconstruction is achieved for imaging an irregular and extended object with a heterogeneous perfusion pattern. The improvement is visible especially in brain regions with higher UR’s and AI’s, where a superior system sensitivity is required in combination to a high spatial resolution.

Similarly, the results from the low-contrast focal lesion detectability phantom (Fig. 11) confirmed the enhanced imaging performance offered by non-conventional apertures. The performances attainable with loftholes, micro-slit and micro-ring apertures are comparable for lesions with bigger diameters (7 and 8 mm), where no collimator recovers the full contrast (CRC 100%), but all five reach the phantom UR. On the other hand, micro-slit and micro-ring apertures outperform the lofthole collimators in following the variation and quantifying the activity concentration and the contrast in lesions with smaller diameters (5 mm).

It is worth noting that the four focal lesions are all located outside or on the border of the higher sensitivity region of the micro-ring geometry. Therefore, we would expect to see further improved performance from the micro-ring apertures when the dimensions of the micro-ring openings are adjusted to provide a large focal region covering the lesions. Secondly, the random distribution of the micro-slit orientations may not be an optimal design choice, which would require further optimization. Nevertheless, the reported results from the 150 *μ*m micro-slit show already some promising benefits over the 1-mm D lofthole, such as a higher intrinsic spatial resolution (Fig.5), higher CNR and CRC values (Fig. 8-9), more accurate UR and AI estimations (Fig.10), and higher lesion detectability (Fig. 11). We plan to address these topics in our future design studies.

It is worth mentioning that the real-world detection system may suffer from physical imperfections, such as charge-sharing and charge trapping, which were not simulated in the current work, but would reduce the effective sensitivity of the system from the simulated values. Further experimental work is planned to assess this impact.

Finally, the simulated imaging studies demonstrate that the SCE camera design assures sufficient projection view-angles over the clinically relevant FOV using a stationary system. From a clinical perspective, this would facilitate dynamic imaging of seizure foci located in both mesial-temporal and extra-temporal locations for MIFE applications.

## V. Conclusion

We have presented the design of a high-resolution and high-sensitivity stationary brain SPECT system, the HelmetSPECT system, based on SCE collimator and modular solid-state detectors. We have designed and evaluated different apertures having non-conventional shapes, namely micro-slit and micro-ring, and compared their performance to the traditional lofthole geometry.

To demonstrate the effectiveness and advantages of the proposed system geometry and novel collimators, we have carried out a series of simulation imaging studies using a Defrise phantom, a hot-rod phantom, an ictal brain perfusion phantom, and a low-contrast focal lesion detectability phantom. The simulated SPECT images obtained from micro-slit and micro-ring apertures showed a superior performance in terms of imaging resolution and contrast recovery in images reconstructed from both noiseless and noisy projection data.

Note that high-performance CZT detectors as simulated in this work are currently associated with a relatively high production cost. Further refinement of CZT crystal growth techniques and detector fabrication process would be needed to make them more cost-effective for a widespread use. Conventional NaI(Tl) detectors currently provide a reasonable compromise between cost and system performance for brain SPECT applications.

The current results demonstrated that the proposed HelmetSPECT system, especially the combination of non-conventional micro-slit and micro-ring apertures with the SCE camera design, could potentially allow for ultrahigh resolution and ultrahigh sensitivity in dynamic imaging of medically intractable epilepsy. Further experimental work will be carried out to confirm these findings.

## Supplementary Material

supp1-3096920
