# Supplementary material for: Design Study of an Ultrahigh Resolution Brain SPECT System Using a Synthetic Compound-Eye Camera Design With Micro-Slit and Micro-Ring Apertures
Source: IEEE Trans Med Imaging. Author manuscript; Available in PMC 2021 Dec 27. (PMC8711775; doi:10.1109/TMI.2021.3096920)
Supplement: supp1-3096920 [file NIHMS1760780-supplement-supp1-3096920.pdf]

Supporting Document to Design Study of an Ultrahigh Resolution Brain SPECT System Using a Synthetic Compound-Eye Camera Design with Micro-Slit and Micro-Ring Apertures

TABLE I
HELMETSPECT BASIC SPECIFICATIONS

PARAMETER	VALUE
Total number of detectors	502
Total number of apertures	502
Total number of independent rings	18 divided in 11 (hemisphere), 7 (neck section)
Number of detectors in each ring	1 (top cover), 5, 12, 18, 24 ,30 ,34, 39, 42, 45, 47, 48, 25, 25, 24, 23, 22, 20, 18
Single detector dimensions	20 mm × 20 mm x 5 mm (thick)
Detector Pixel array	80 × 80
Detector Pixel size	250 µm × 250 µm
Sphere radius*	196 mm
Object-space radius	100 mm
Detector polar angle	$0 \leq \theta_m < ¾ \pi$
Detector azimuthal angle	$0 \leq \varphi_m < 2\pi$ in the hemispherical section,<br>$0 \leq \varphi_m < \pi$ in the neck section

* defined as the distance from the center of the sphere to the front surface of the detector.

TABLE II
BASIC SPECIFICATIONS OF THE COLLIMATOR APERTURES

PARAMETER	PINHOLE		MICRO-SLIT		MICRO-RING
Total number of apertures	502		502		502
Dimensions	(502×) 1 mm D	(168×) 500 µm D, (167×) 1.5 mm D, (167×) 3 mm D	W × L: 250 µm × 5 mm	W × L: 150 µm × 6 mm	Outer radius 7.5 mm width 250 µm
MF(s)	1:12		1:12		1:12
Acceptance angle α	27.3°		27.3° (1)<br>35.3° (2)		27.3°
Exit angle β	29.4°		29.4° (1)<br>22.6° (2)	29.4° (1)<br>21.1° (2)	29.4°
Thickness upper profile	10 mm		11 mm		7 mm
Thickness lower profile	10 mm		9 mm		13 mm
Material	Tungsten		Tungsten		Tungsten

(1) Short direction
(2) Long direction

TABLE III
BRAIN REGIONS AND MAIN PARAMETERS

Functional VOI	*ABBREVIATION*	*VOLUME (cm³)*		*PHANTOM UR(2)*		*PHANTOM AI (3)*
		LEFT	RIGHT	LEFT (ICTAL)	RIGHT (NORMAL)	
White Matter	WM	258.424	254.104	0.6883	0.6967	-0.0120
Cerebellum	CER	73.024	72.936	1.0533	1 (1)	0.0533
Caudate-Nuclei	CN	4.904	5.192	0.9333	0.9417	-0.0088
Lenticular-Nuclei	LN	6.328	6.864	1.1217	1.0267	0.0925
Thalamus	TH	5.976	5.92	1.0983	1.025	0.0715
Insula	IN	6.536	5.88	1.175	0.9850	0.1929
**Temporal-Pole**	**TPO**	**1.424**	1.632	**1.2483**	0.9083	**0.3743**
Temporal-Mesial	TM	5.24	5.408	1.055	0.9833	0.0729
Temporal-Lateral	TL	91.152	88.976	1.1	0.89	0.2360
Temporal-Posterior	TP	24.712	21.592	0.855	0.87	-0.0172
Frontal- Pole	FRPO	2.48	2.48	0.7750	0.8167	-0.0510
Frontal-Orbit	FRO	24.744	26.344	1.1833	1.005	0.1774
Frontal-Mesial	FRM	60.944	62.36	0.8	0.8233	-0.0283
Occipital-Mesial	OCCM	11.92	12.8	0.9783	1.0667	-0.0828
Occipital-Lateral	OCCL	29.896	27.128	0.6633	0.6267	0.0585
Parietal	P	63.44	63.3	0.7467	0.7883	-0.0529

(1) Cer-R is used as reference region for UR calculation.
(2) The uptake ratio is defined in (10)
(3) The asymmetry ratio is defined in (11)

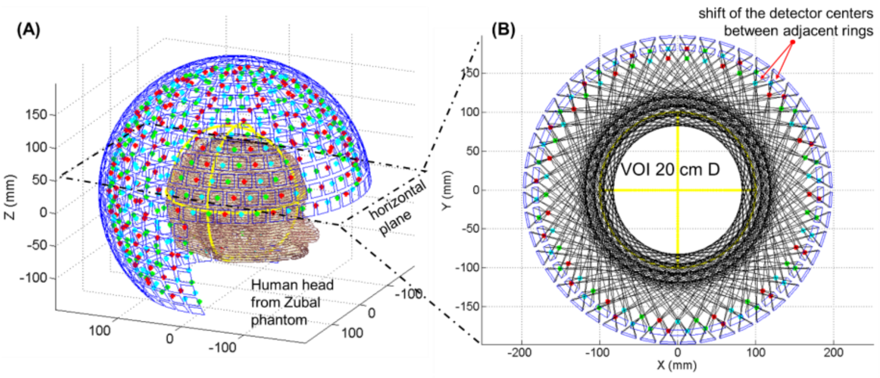

Fig.1S HelmetSPECT geometry with lofthole apertures: **(A)** complete view with distribution of the 3 types of loftholes**; (B)** angular sampling in the horizontal plane. Legend: blue line-detector surface, red dot-500 µm-D lofthole center, green dot- 1.5 mm-D lofthole center, cyan dot- 3mm-D lofthole center, yellow sphere- object space 20 cm in diameter, pink contour lines- human head, black line in (B) connects the right (/left) corner in the upper profile of the insert to left (/right) corner in the lower profile and is extended to the detector surface and object space. In every ring of the spherical design, all three types of lofthole are used, having each lofthole different from the previous and the following in the same ring.

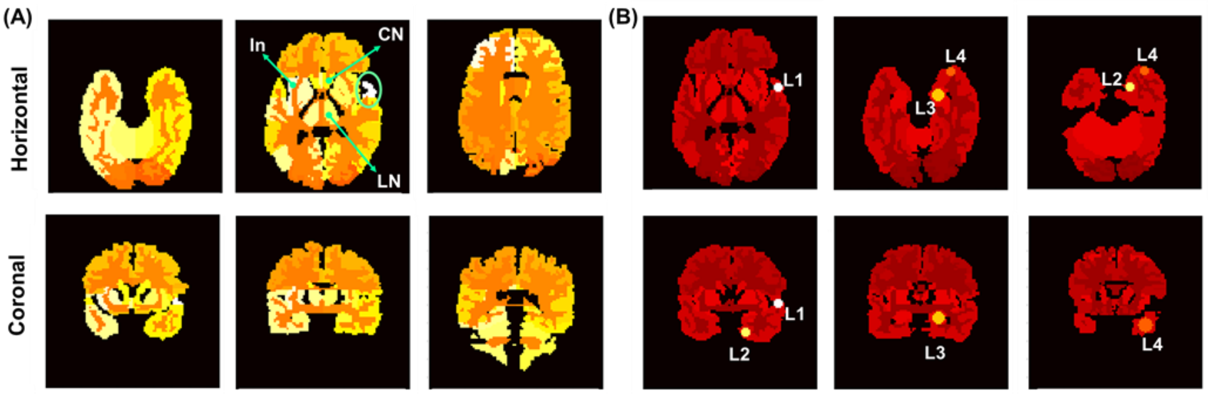

Fig.2S Horizontal and coronal planes of **(A)** the MTLE ictal perfusion brain phantom and **(B)** the low-contrast focal lesion detectability phantom (L1-lesion 1 contrast 3:1 and diameter 5 mm, L2-lesion 2 contrast 2.5:1 and diameter 6 mm, L3-lesion 3 contrast 2:1 and diameter 7 mm, L4-lesion 4 contrast 1.5:1 and diameter 8 mm). Both phantoms have 96×96×96 voxels with isotropic 2 mm ×2 mm ×2 mm voxel size. In (A), the TPo region is circled in green, and the internal areas of CN, LN and In are shown.

DIGITAL BRAIN PHANTOM

The *ictal perfusion phantom* (Fig.2S(A)) was derived according the procedure described by Grova et al. in [1]. The digital Zubal phantom [2] was used as spatial model for the brain anatomy. The original phantom consists of a 256×256×128 matrix with anisotropic voxel 1.1 mm ×1.1 mm ×1.4 mm, segmented and labelled in 62 anatomical structures or VOIs. In MATLAB, we applied a cubic interpolation to the original phantom to obtain a 96×96×96 matrix with isotropic 2 mm ×2 mm ×2 mm voxel size. The anatomical asymmetry between the left and right hemispheres in the Zubal phantom was not removed. The original 62 VOIs were further segmented or combined to generate 16 VOIs (listed in Table III) needed in the spatial model of MTLE ictal brain anatomy. We will refer to these 16 VOIs as *functional VOIs*. Lastly, the 16 functional VOIs were lateralized for the left and right hemisphere after defining the inter-hemispheric plane, for a total of 32 *anatomical VOIs*. The lateralization is needed due to the asymmetric perfusion values between the healthy (right) and ictal (left) hemisphere, typical in MTLE. The activity distribution map was then generated according the perfusion values reported in Table 3 in [1]. The empirical average activity values $\bar{x}_\iota$ in the left (ictal) hemisphere ($\overline{x_{\iota L}}$) and the right (healthy) hemisphere ($\overline{x_{\iota R}}$) were assigned to the corresponding VOI *i* (*i*∈[1, 32]) in the digital phantom (Fig.2S).

The *low-contrast focal lesion detectability phantom* (Fig.2S(B)) was derived from the ictal perfusion phantom described above, using the healthy perfusion values (third column in Table 3 in [1]) for both the left and right hemisphere. Four spherical simulated lesions with diameters of 5, 6, 7 and 8 mm were included in the non-lateralized perfusion phantom. The low lesion-to-background contrast levels were set as 3:2.5:2:1.5:1 for lesion1:lesion2:lesion3:lesion4:background where the background is the white matter. The lesion-to-background contrast decreases with the increasing lesion diameter.

[1] C. Grova *et al.*, "A methodology for generating normal and pathological brain perfusion SPECT images for evaluation of MRI/SPECT fusion methods: application in epilepsy," *Physics in Medicine and Biology,* vol. 48, no. 24, pp. 4023-4043, 2003/12/05 2003, doi: 10.1088/0031-9155/48/24/003.

[2] I. G. Zubal, C. R. Harrell, E. O. Smith, Z. Rattner, G. Gindi, and P. B. Hoffer, "Computerized three-dimensional segmented human anatomy," *Med Phys,* vol. 21, no. 2, pp. 299-302, 1994, doi: 10.1118/1.597290.

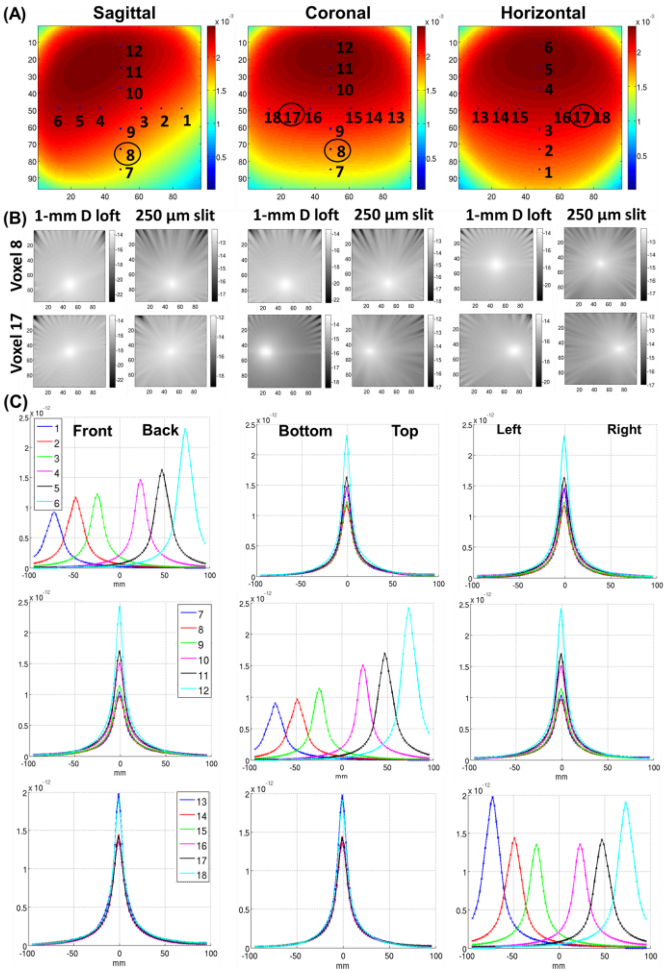

Fig.3S (A) Sensitivity maps for the HelmetSPECT geometry when 250 µm micro-slits are used, overlapped to the locations of 18 out-of-center voxels in the FOV where the FIM image is estimated. The voxels are placed at 2.5 cm, 5 cm and 7.5 cm away from the center along the cranio-caudal, lateral and ventro-dorsal directions. **(B)** Comparison between the out-of-center FIM images of the 8th (first row) and 17th (second row) voxel for the 1-mm D lofthole and 250 µm micro-slit in the sagittal, coronal, and horizontal planes. **(C)** 1D profiles from the target voxels in (A) along the ventrodorsal (first column), craniocaudal (second column), and lateral (third column) directions across the object space.

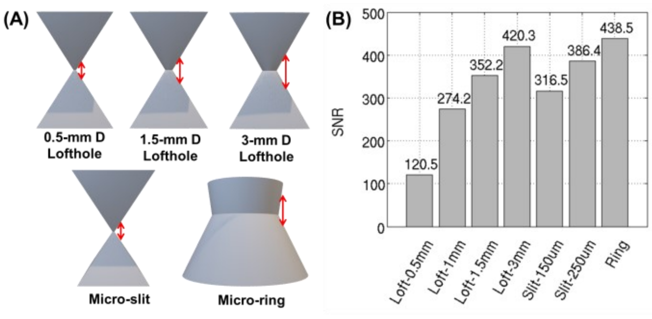

Fig.4S (A) 3D drawings of the aperture inserts showing the different thicknesses of the collimator material close to the insert opening. **(B)** Aperture SNR values according (13).

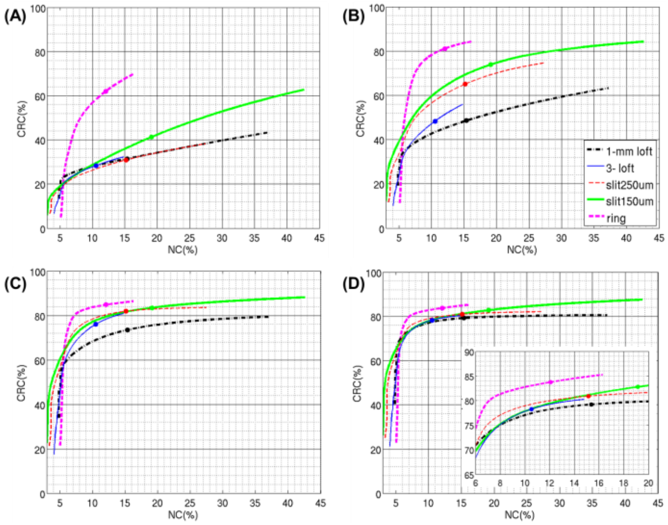

Fig.5S CRC-NC plots from the five simulated collimators of: **(A)** 4-mm D, **(B)** 6-mm D, **(C)** 8-mm D and **(D)** 10-mm D hot rod from the resolution phantom in Fig.9 in the full range of iterations. The dots show the iteration with lowest NRMSE (same iteration shown in Fig.9). (D) includes a zoomed view of the curves.

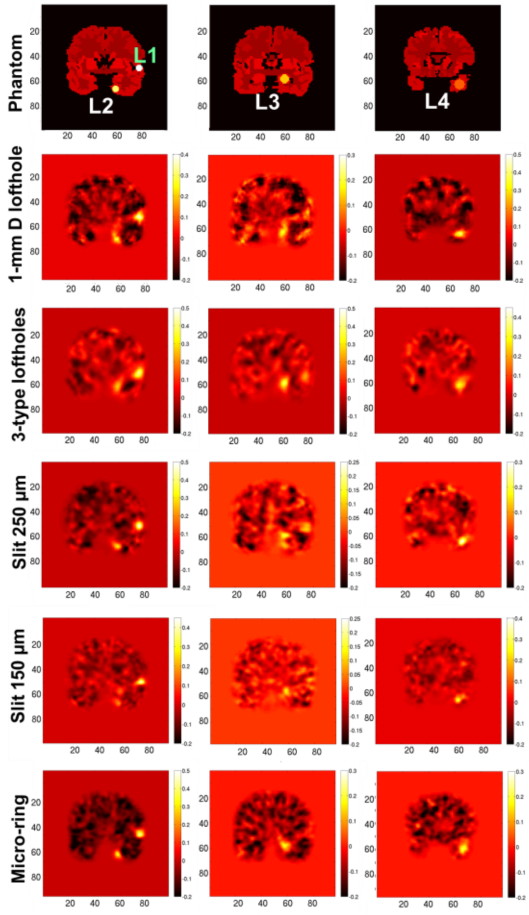

Fig.6S Subtraction images from the low-contrast focal lesion detectability brain phantom at the iteration with lowest NRMSE, coronal plane: **(first row)** digital phantom, **(second row)** 1-mm D lofthole (10th iteration), **(third row)** 3-type lofthole (21st iteration), **(fourth row)** micro-slit 250 µm× 5 mm (8th iteration), **(fifth row)** micro-slit 150 µm× 6 mm (5th iteration), **(sixth row)** micro-ring (27th iteration). The constructed images are filtered with a 3D 6-mm FWHM Gaussian filter and have 2-mm slice thickness. The images are shown according to the radiological display convention.
